# Structural and Biochemical Insights into Post-Translational Arginine-to-Ornithine Peptide Modifications by an Atypical Arginase

**DOI:** 10.1021/acschembio.2c00879

**Published:** 2023-02-15

**Authors:** Silja Mordhorst, Thomas Badmann, Nina M. Bösch, Brandon I. Morinaka, Hartmut Rauch, Jörn Piel, Michael Groll, Anna L. Vagstad

**Affiliations:** † Institute of Microbiology, Eidgenössische Technische Hochschule (ETH) Zürich, Vladimir-Prelog-Weg 4, 8093 Zürich, Switzerland; ‡ Chair of Biochemistry, Center for Protein Assemblies, Technical University of Munich, Ernst-Otto-Fischer-Str. 8, 85748 Garching, Germany

## Abstract

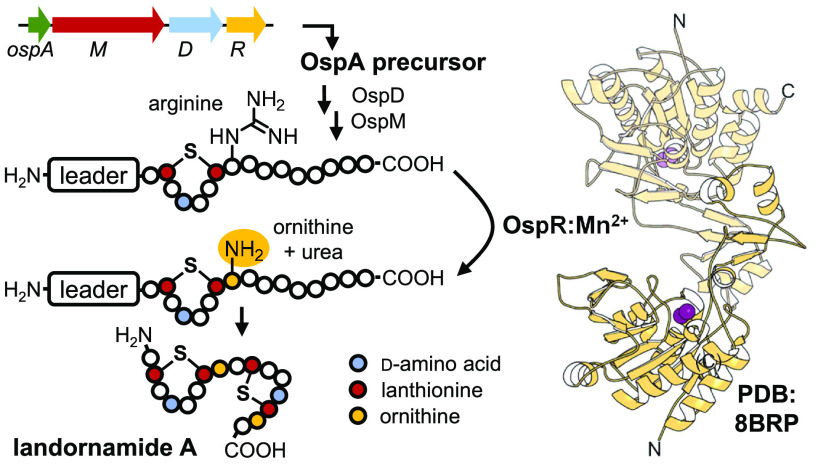

Landornamide A is a ribosomally synthesized and post-translationally modified peptide (RiPP) natural product with antiviral activity. Its biosynthetic gene cluster encodes—among other maturases—the peptide arginase OspR, which converts arginine to ornithine units in an unusual post-translational modification. Peptide arginases are a recently discovered RiPP maturase family with few characterized representatives. They show little sequence similarity to conventional arginases, a well-characterized enzyme family catalyzing the hydrolysis of free arginine to ornithine and urea. Peptide arginases are highly promiscuous and accept a variety of substrate sequences. The molecular basis for binding the large peptide substrate and for the high promiscuity of peptide arginases remains unclear. Here, we report the first crystal structure of a peptide arginase at a resolution of 2.6 Å. The three-dimensional structure reveals common features and differences between conventional arginases and the peptide arginase: the binuclear metal cluster and the active-site environment strongly resemble each other, while the quaternary structures diverge. Kinetic analyses of OspR with various substrates provide new insights into the order of biosynthetic reactions during the post-translational maturation of landornamide A. These results provide the basis for pathway engineering to generate derivatives of landornamide A and for the general application of peptide arginases as biosynthetic tools for peptide engineering.

Peptide arginases are a recently discovered enzyme family (pfam12640) catalyzing the hydrolysis of arginine residues incorporated in a peptide chain to ornithine (Orn) units, constituting an unprecedented post-translational modification.^1^ These enzymes occur in biosynthetic pathways of ribosomally synthesized and post-translationally modified peptide (RiPP) natural products. RiPPs are built from gene-encoded precursor peptides typically composed of an N-terminal leader and a C-terminal core peptide. The core is subject to modification by post-translational maturases, after which the leader is released by a protease to yield the mature RiPP. The first characterized peptide arginase representative is OspR (OSCI_RS22075) from the cyanobacterium *Kamptonema* sp. (formerly *Oscillatoria*) PCC 6506. OspR hydrolyzes two arginine residues to ornithines in the precursor peptide OspA during the biosynthesis of landornamide A (Figure 1A–C), a member of the proteusin family of RiPPs with antiviral activity toward lymphocytic choriomeningitis virus.^2^ Landornamide A is formed in six post-translational modification steps: two lanthionine bridges installed by OspM, two C_α_ epimerizations catalyzed by the peptide epimerase OspD, and two arginine-to-ornithine hydrolyses by OspR. Preliminary heterologous pathway expression experiments in *Escherichia coli* indicated that the activity of the lanthionine synthetase OspM is dependent on the installation of d-amino acids by the radical *S*-adenosylmethionine peptide epimerase OspD. In contrast, OspR converts both arginine residues of the OspA core peptide in a manner independent of the modifications installed by OspM and OspD.^2^ Therefore, the biosynthetic order of reactions remained to be determined.

**Figure 1:**
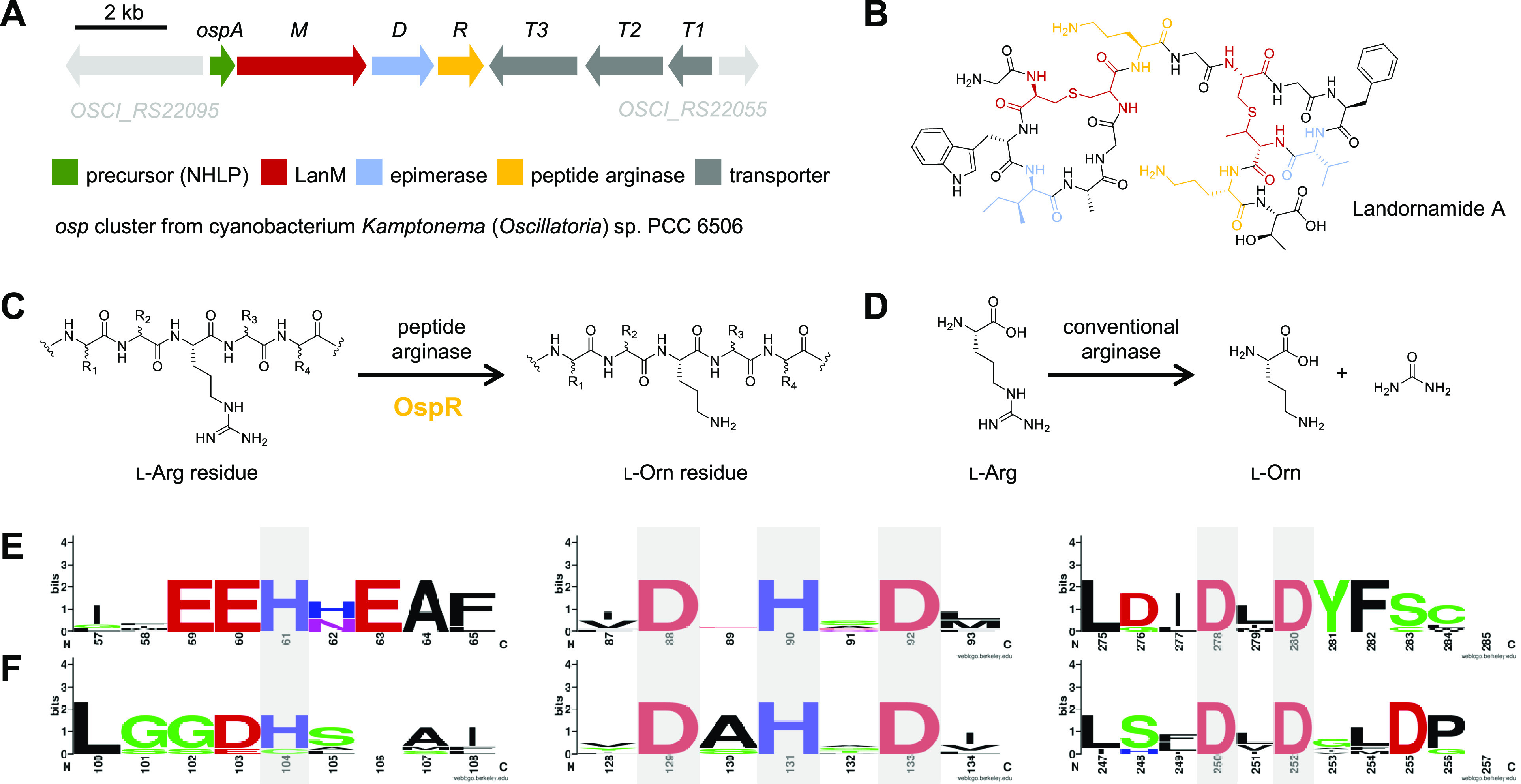
Peptide arginase OspR post-translationally installs ornithines into the precursor protein OspA during landornamide A biosynthesis. (A) Biosynthetic gene cluster and (B) chemical structure of landornamide A. Post-translational modifications are colored to match the responsible maturase enzymes. Generic enzymatic reaction of (C) peptide arginases and (D) conventional arginases. Conserved sequence motifs of (E) peptide arginases and (F) conventional arginases. The sequence motifs were built with WebLogo,^12,13^ based on the arginase sequence alignments from Figures S3 and S4. The six residues shaded in gray coordinate metals in the active sites.

Peptide arginases are weakly similar to the well-characterized family of conventional arginases (EC 3.5.3.1, also known as l-arginine amidinohydrolases) that hydrolyze free l-arginine monomers to l-ornithine and urea (Figure 1D). Conventional arginases are found in all three domains of life and have been studied extensively.^3,4^ Two isoforms of this enzyme exist in humans: arginase-1 is mainly found in the cytoplasm of liver cells and catalyzes the last step of the urea cycle to metabolize ammonia, while arginase-2 is found in the mitochondria where it regulates nitric oxide levels and the ratio of intracellular arginine to ornithine.^3^ In bacteria, arginases catalyze the first step of the arginase pathway, which eventually yields two molecules of glutamate. Bacterial and fungal arginases play roles in basic nitrogen metabolism and nitrogen redistribution. They produce l-ornithine, which serves as a precursor for l-proline, polyamines, and larger specialized metabolites such as antibiotics. Moreover, they are involved in stress resistance and pathogenesis; i.e., the arginase RocF is a key virulence factor in *Helicobacter pylori*.^4−6^

Arginases are metalloenzymes and belong to the ureohydrolase superfamily. They contain three conserved sequence motifs: GGDHS, DAHXD, and SXDXDXXDP (Figure 1E). The six underlined residues coordinate a catalytically relevant binuclear manganese cluster at the active site. Mechanistic studies for the mammalian arginases from rat and human and the bacterial arginase from *Bacillus caldovelox* have been published.^7−10^ The common mechanistic proposal includes metal-activated hydroxide formation by deprotonation of water followed by nucleophilic attack of the hydroxide at the carbon atom of the positively charged guanidino group of the substrate arginine. In the tetrahedral intermediate, a proton is transferred from the nucleophile via an aspartate to the ε-nitrogen atom of the leaving group ornithine. The liberation of ornithine and the formation of urea are the last steps before addition of water to the metal cluster for the next catalytic cycle.^8,10,11^

In this study, we established in vitro activity for OspR, enabling the first biochemical and structural characterization of a peptide arginase. Kinetic analyses of OspR toward differentially modified OspA precursors revealed the preferred order of post-translational modifications during the biosynthesis of the antiviral compound landornamide A. Cumulative results rationalize the substrate preferences of peptide arginases and will inform future engineering efforts for ornithine-containing peptides.

## Results and Discussion

The aim of this work was to understand at a molecular level the formation of ornithine residues in a peptide chain as a novel post-translational modification. Our study combines aspects of chemistry, structural biology, and biochemistry to investigate the enzymatic reaction from peptidyl arginine to ornithine and urea, elucidate the enzyme crystal structure, and establish the biosynthetic timing of ornithine formation in landornamide A biosynthesis through detailed in vitro experiments.

### Sequence Analysis of Peptide Arginases

OspR and the other bacterial peptide arginases identified in a bioinformatics analysis of RiPP biosynthetic gene clusters^1^ are members of the protein family pfam12640 that also contains the human protein C5orf22. This protein of unknown function has been implicated in the regulation of mRNA splicing.^14^ OspR shares a higher sequence similarity with C5orf22 (32.2%, Figure S1) than with conventional arginases (e.g., 20.9% to human arginase-1), and we hypothesize that C5orf22 functions as a peptide arginase. Human arginase-1 shows 60–93% similarity to other conventional arginases including bacterial representatives, whereas pfam12640 family members are more divergent. OspR exhibits only 41–62% similarity to other verified bacterial peptide arginases (Table S1). Regions of relative sequence conservation and variability of peptide arginases are depicted in Figure S2. Multiple sequence alignments suggest that peptide arginases retain the metal-coordinating Asp and His residues observed in conventional arginases, but they are embedded in three alternative conserved active-site motifs (Figures S3 and S4). Bacterial peptide arginases harbor a central DXHXD motif with less conservation of the second position relative to the analogous motif of conventional arginases, DAHXD. The two other sequence motifs of peptide arginases EEH(N/H)EAF and LDIDLDYFSC substitute for the GGDHS and SXDXDXXDP in conventional arginases, respectively (Figure 1E,F). The role of these sequence motifs will be discussed further in the Structural Characterization of OspR section.

### Biochemical Characterization of OspR In Vitro

OspR was heterologously produced in *E. coli* as a solubility-tag fusion with N-terminal His_6_-tagged maltose binding protein (MBP) due to the low production and instability of other constructs. Following protein purification by Ni^2+^-affinity chromatography, attempts to remove the MBP-tag by digestion with the tobacco etch virus protease (TEVp) resulted in only incomplete cleavage products. Therefore, biochemical analyses were performed with the as-purified His_6_-MBP-OspR fusion, referred to here as OspR. His_6_-tagged OspA precursor protein substrates were produced as previously described (Figure S5).^15^

**Figure 2:**
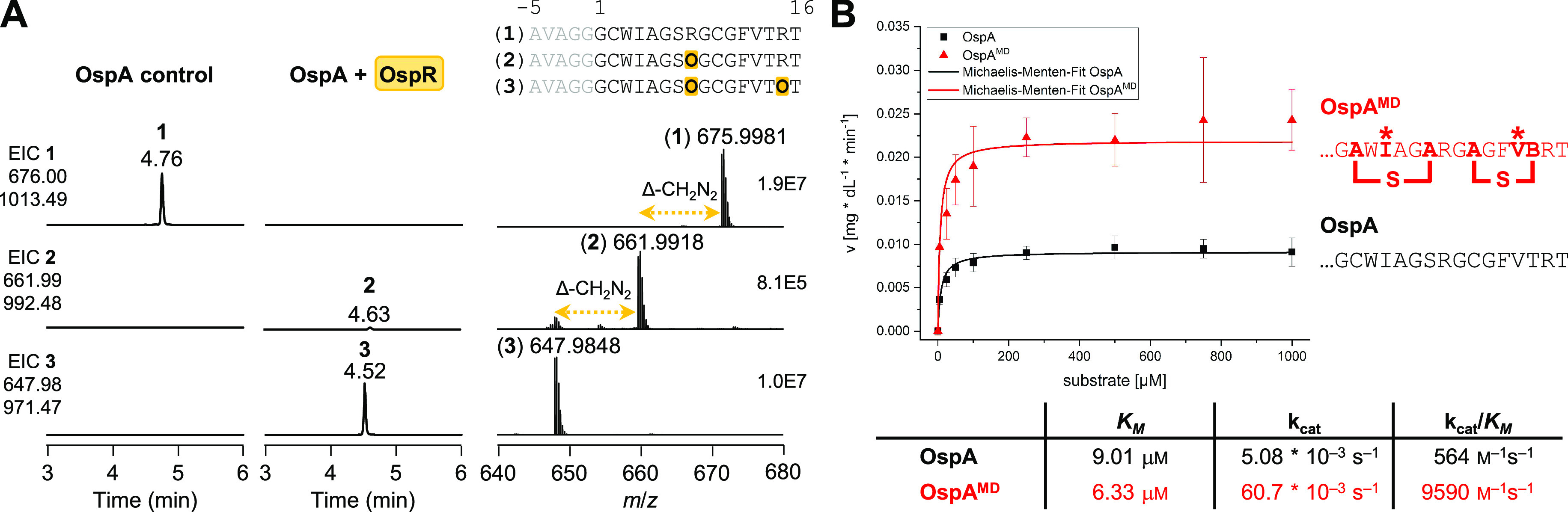
In vitro reactions with OspR. (A) LC-HRMS of OspA reaction products relative to the control lacking OspR. Extracted ion chromatograms (EICs) for the predicted [M + 3H]^3+^ and [M + 2H]^2+^ ions for the unmodified **1**, single-modified **2**, and double-modified **3** GluC-released core peptide fragments. Mass spectra for the relevant [M + 3H]^3+^ ions with monoisotopic masses are shown to the right. Hydrolysis of arginine to ornithine and urea accounts for the observed deconvoluted mass loss of 42.02 Da (−CH_2_N_2_) per ornithine. (B) Michaelis–Menten kinetic measurements by urea detection assay for unmodified OspA and cyclized-and-epimerized OspA^MD^ substrates. *, d-amino acid; B, butyrine residue.

In an initial set of experiments, the ability of OspR to convert the Arg8 and Arg15 residues in the core sequence of OspA to the expected ornithine and urea products was monitored by two assays: Peptide products were detected by liquid chromatography-high resolution mass spectrometry (LC-HRMS) following proteolytic release of the OspA_-5-16_ core peptide fragment from His_6_-OspA (12.8 kDa) by digestion with glutamyl endoprotease (GluC). Urea was detected by a commercially available end-point spectrophotometric assay monitoring urea formation at 430 nm.^16^ Results of the in vitro assays paralleled previously published in vivo coexpression experiments.^1^ In an initial in vitro reaction with OspR, the unmodified OspA substrate (detected as core peptide **1** following GluC cleavage) was converted to the Arg8Orn intermediate (**2**) and the Arg8Orn–Arg15Orn product (**3**) with mass losses of 42.02 and 84.04 Da, respectively, relative to the control lacking OspR (Figure 2A). As expected, the spectrophotometric assay confirmed urea as the coproduct of peptide arginases, like for conventional arginases.

For in vitro reaction rate experiments, where a higher sample throughput was desired, the urea detection assay in 96-well-plate format was chosen to monitor the activity of OspR. First, various in vitro reaction conditions (with permutations in the buffer composition, pH, temperature, and manganese concentration) were screened to identify assay conditions that supported efficient turnover of OspA (Figure S6). The optimal conditions (50 mM TRIS-HCl pH 8.5, 1 mM MnCl_2_, 25 °C) were used for all further experiments. We additionally tested if OspR could accept free l-arginine or the peptidyl-arginine mimetic *N*-acetylarginamide, but no urea formation was detected (Figure S7), suggesting that OspR requires a peptide chain as the substrate.

Next, a Michaelis–Menten kinetic analysis of OspR toward OspA was conducted (Figures 2B and S8). The calculated constants differed substantially from those reported for conventional arginases. OspR is considerably slower, with a *k*_cat_ of only 5.08 × 10^–3^ s^–1^ compared to 300 s^–1^ for human arginase-1 as a representative example.^17^ This is consistent with the generally slower kinetic rates of secondary metabolic versus primary metabolic processes.^18^ OspR appears to bind its peptide substrate with higher affinity than conventional arginases with a *K*_M_ of 9.01 μM relative to 2.3 mM reported for human arginase-1 toward arginine.^17^ This may be reflective of the more extensive protein–protein interactions possible between OspR and OspA.

### Biosynthetic Timing of Ornithine Modifications in Landornamide A Biosynthesis

To date, our previous studies could not resolve the biosynthetic timing of arginine-to-ornithine modifications by OspR during landornamide A biosynthesis. Previous heterologous coexpression experiments with the complete biosynthetic cassette (i.e., OspAMDR), in which single enzyme functions were individually blocked by inactivating mutations, only yielded OspA core peptide products with complete conversion of arginines to ornithines when functional OspR was present.^2^ To test if OspR accepts modified substrates in vitro, the fully cyclized-and-epimerized OspA precursor protein with (methyl)lanthionine bridges installed at Cys2–Ser7 and Cys10–Thr14 and with d-amino acids at Ile4 and Val13 was produced by coexpression of *ospA* (His_6_-tagged) with *ospM* and *ospD*. The resulting OspA^MD^ variant was confirmed to be a substrate by urea detection assay and was similarly subjected to Michaelis–Menten kinetic analysis for comparison to the unmodified OspA substrate (Figure 2B, Figure S8, and Table S2). The catalytic efficiency (*k*_cat_/*K*_M_) is 17-fold higher for the modified OspA^MD^ compared to the unmodified OspA substrate. This difference is mostly accounted for by the 12-fold higher turnover number measured for OspA^MD^ (*k*_cat_ 60.7 × 10^–3^ s^–1^) relative to linear OspA (*k*_cat_ 5.08 × 10^–3^ s^–1^), whereas the *K*_M_ values are similar at 6.33 and 9.01 μM, respectively. Thus, the preferred substrate of OspR contains cyclized and/or epimerized residues.

**Figure 3:**
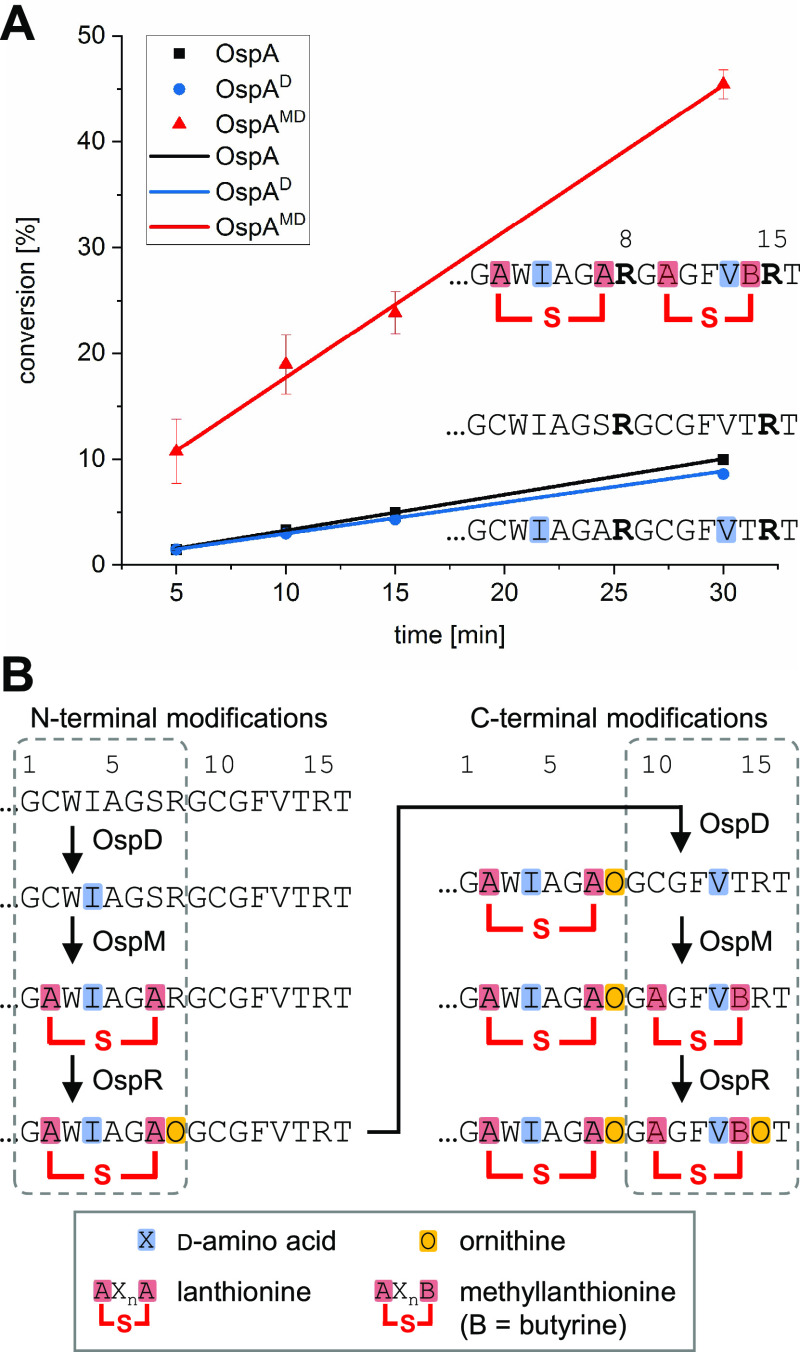
Biosynthetic timing of post-translational modifications in landornamide A. (A) Relative rate comparison of arginine-to-ornithine conversions in OspA substrate variants catalyzed by OspR. The core structures are shown next to their plot with the substrate Arg8 and Arg15 residues indicated in bold. Error bars represent standard deviation of triplicate timepoints. (B) Revised proposal for maturation of the OspA precursor protein. The OspD epimerase, OspM lanthionine synthetase, and OspR arginase preferentially act in a stepwise fashion to modify the N-terminal region prior to the C-terminal region.

To gather further evidence of the favored route to landornamide A, we measured the relative rate of OspR toward differentially modified OspA substrates by the LC-HRMS assay so that modification at Arg8 and Arg15 could be distinguished (Figure 3A). We produced three OspA variants as test substrates for OspR: the (i) unmodified (“OspA”) and (ii) cyclized–epimerized (“OspA^MD^”) versions already tested in the urea detection assay, as well as (iii) epimerized only (“OspA^D^”) produced by coexpression of *ospA* with *ospD*. These expressions resulted in the desired Ni^2+^ affinity-purified OspA variants in high purity (Figures S5 and S9). The core peptide sequences of the substrate, intermediate, and product contain zero, one, and two ornithines, respectively, and were detected by LC-HRMS from triplicate in vitro reactions with OspR (Figure S10). Relative rates were calculated for early timepoints in the linear range of ornithine formation. The percent conversion data is based on the relative peak areas for the EICs of all core peptides weighted by the number of ornithines, as established in our previous study.^1^

Fully cyclized–epimerized OspA^MD^ was converted most rapidly, and this reaction was set as the benchmark for rate comparisons (Figure S10). The two linear substrates, unmodified OspA and epimerized OspA^D^, were converted at 24 and 21% rate of OspA^MD^, respectively (Table S3). Furthermore, additional digestion of selected samples with trypsin protease (in which ornithine residues are resistant to proteolysis resulting in missed cleavage sites) and tandem mass spectrometry (MS^2^) analysis allowed us to interpret which of the two arginines was favored by OspR (Figures S11–S13 and Table S4). In the two linear substrates, Orn8 was the only detected intermediate, consistent with N- to C-terminal core processing, as also observed for the established in vivo conversions.^1^ As the exception, cyclized–epimerized OspA^MD^ formed both of the possible single ornithine-containing intermediates: Orn8 as the major and Orn15 as the minor species, suggesting modifications adjacent to (methyl)lanthionine rings can partially override the preferred directionality.

Prior experimental results from full-cluster^2^ and OspR^1^ coexpressions in *E. coli* and in vitro analysis of OspD^15^ demonstrated that the OspA core is processed in an N- to C-terminal direction by all maturases in the pathway. Furthermore, the activity of OspM was dependent on the presence of d-amino acids, suggesting that epimerization at Ile4 occurs prior to formation of Cys2–Ser7 lanthionine.^2^ Taking into account these cumulative results with our new understanding that OspR acts after installation of the adjacent d-amino acid and lanthionine bridge, we propose that OspA is preferentially processed sequentially by OspD, OspM, and OspR at the N-terminal modification sites followed by the C-terminal sites in landornamide A biosynthesis (Figure 3B).

### Metal Dependence

Arginases are metallohydrolases and require two coordinated metal ions to convert their substrates. In order to interrogate the importance of the conserved metal-binding DXHXD sequence motif, three OspR variants were cloned: the two single mutants, Asp43Ala and Asp43Asn, and the triple mutant Asp39Asn-His41Gln-Asp43Asn. For all three variants, in vitro reactions with unmodified OspA as the substrate were followed by urea detection assay against controls. Wildtype OspR served as a positive control. An aliquot of this protein preincubated with the metal chelator ethylenediaminetetraacetate (EDTA) served as a negative control. EDTA treatment was previously shown to inactivate conventional arginases by removing the essential binuclear manganese cluster.^19^ Relative urea production was compared for the 4 h reaction timepoints. Compared to the wildtype enzyme, the activity of all three mutants was significantly reduced: OspR-D43A and OspR-D43N showed 14 and 5% relative activity, respectively, while the activity of the triple mutant OspR-D39N-H41Q-D43N was comparable to the EDTA-treated negative control, at around 1% for both samples (Figure S14). These results are consistent with studies on conventional arginases bearing point mutations in this sequence motif^11,20,21^ and indicate that these conserved residues are essential for peptide arginase catalysis.

Conventional arginases are mostly manganese-dependent enzymes.^20,22^ We next performed inductively coupled plasma-mass spectrometry (ICP-MS) analysis on OspR purified from *E. coli* to confirm that manganese is also naturally chelated by peptide arginases (Tables S5–S6, Figure S15). In comparison, bound manganese was depleted in the EDTA-treated wildtype OspR sample (11.7%) and the DXHXD motif triple mutant sample (1.8%) relative to the as-purified wildtype OspR. As expected, EDTA treatment reduced activity of OspR by ∼99% (Figure S14), confirming that the metal cluster is crucial for enzymatic activity.

Metals are installed in metalloenzyme active sites through metal delivery systems or competition from buffered metal pools.^23^ Some conventional arginases accept different metals as a replacement for manganese. Ni^2+^ and Co^2+^, for example, are accepted by human and rat arginase-1 and the arginase from *B. anthracis*,^8,19,21,24,25^ whereas some arginases, such as the arginase from *Zymomonas mobilis* ZM4,^26^ are highly specific for Mn^2+^. Zinc has been reported to inhibit human arginase-1 by forming a carboxylate–histidine–Zn^2+^ triad involving the catalytically relevant histidine residue.^21^ Therefore, we tested the metal promiscuity of OspR. After standard purification, OspR was treated with EDTA, dialyzed and incubated individually with six different divalent metal ions (i.e., Mg^2+^, Ca^2+^, Cu^2+^, Ni^2+^, Co^2+^, and Zn^2+^), in addition to reintroduction of Mn^2+^ as a positive control and no metal supplementation as a negative control. Relative amounts of the urea coproduct at the 4 h timepoint were compared across samples. Two of the six metals tested can replace Mn^2+^ during catalysis in vitro: Ni^2+^ and Co^2+^ are accepted by OspR, with relative activities of approximately 97 and 82%, respectively, compared to the Mn^2+^ positive control (Figure S16). The EDTA-treated negative control retained approximately 10% relative urea production for this batch of protein, likely due to retention of some bound Mn^2+^ in the OspR active site. In rat arginase-1, metal chelation is known to remove only one of the two active-site Mn^2+^;^27^ thus, we cannot rule out that the restored OspR activity is due to mixed-metal clusters rather than pure Ni^2+^ or Co^2+^ binuclear clusters. The other four metal ions, Mg^2+^, Ca^2+^, Cu^2+^, and Zn^2+^ showed conversions similar to EDTA-treated OspR. These results support that OspR requires divalent metal ions for catalysis, and the naturally bound metals are likely Mn^2+^ ions.

### Structural Characterization of OspR

**Figure 4:**
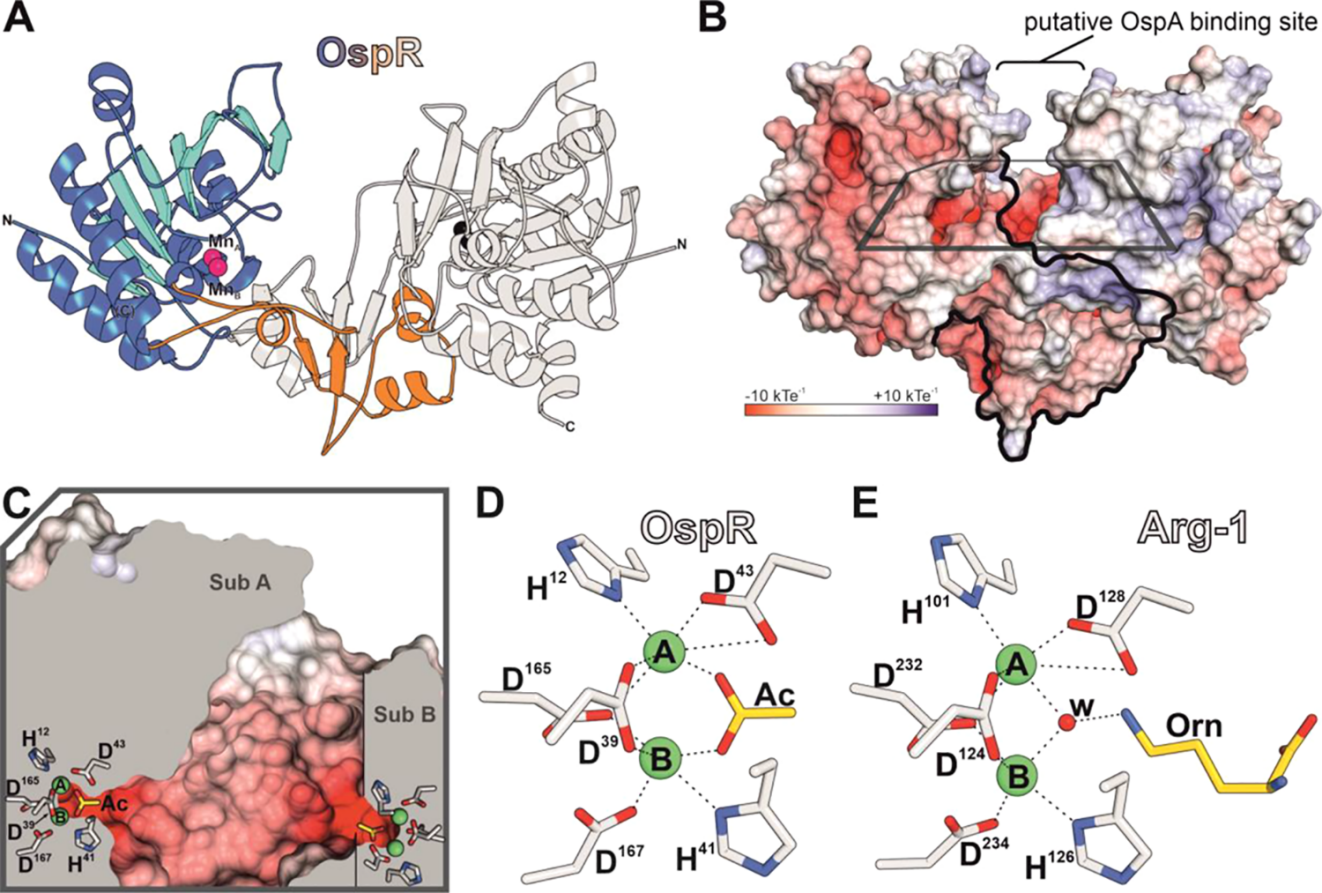
Crystal structure of OspR and active-site comparison with human arginase-1. (A) Cartoon-representation of the OspR homodimer. The protein adopts an α-β-α sandwich fold with an additional dimerization domain (subunit A in color: α-helices and random coils in blue and β-strands in cyan visualize the sandwich fold, manganese in pink, dimerization domain in orange. Subunit B in light gray: manganese in black). (B) Surface charge distribution of OspR with similar orientation as in (A). The border between the two subunits is highlighted by a black line. The putative OspA binding site and active site cleft is marked. (C) Zoomed-in cross-section of the boxed area from (B). The intersection between the two subunits forms a substrate binding site with two accessible active centers, each comprising a binuclear manganese cluster (Mn_A_ and Mn_B_ colored in green). Residues and molecules engaged in manganese binding are depicted as sticks (carbon atoms: gray, oxygen: red, nitrogen: blue) and labeled by one-letter-codes in subunit A. An acetate molecule (Ac, gold) occupies the active site. (D) Ball and stick model of the active site of OspR. Labels and coloring are similar to (C). (E) Active site of human arginase-1 (PDB-ID 3GMZ). An ornithine (Orn, gold) and a water molecule (w, red) occupy the active site.

The ability of OspR to catalyze peptidyl arginine-to-ornithine conversions in contrast to conventional arginases that act on free arginine motivates their comparison from a structural and mechanistic point of view. The comparison is particularly interesting considering the low overall sequence identity of members of these enzyme families (Table S1). An untagged version of OspR was generated by proteolytic removal of an N-terminal His_6_-SUMO tag and was demonstrated to be catalytically competent by LC-HRMS assay (Figure S17). We obtained detailed structural information of untagged OspR by protein X-ray crystallography. Single-wavelength anomalous diffraction techniques allowed determination of the structure to 2.6 Å resolution (PDB-ID 8BRP, Figure 4). A DALI search for structural homologues resulted in several moderately similar structures of members of the arginase family.^28^ The hits include arginases, agmatinases, proclavaminate amidino hydrolases, as well as formiminoglutamases, all of which process substrates featuring a guanidine or formimino moiety.^29^*E. coli* agmatinase is the best structural hit (PDB-ID 7LBA, C_α_-RMSD: 2.9), whereas the closest deposited conventional arginase is from *B. subtilis* (PDB-ID 6DKT, C_α_-RMSD: 3.2). Structures of human arginases have slightly higher C_α_-RMSD values of around 3.3.

**Figure 5:**
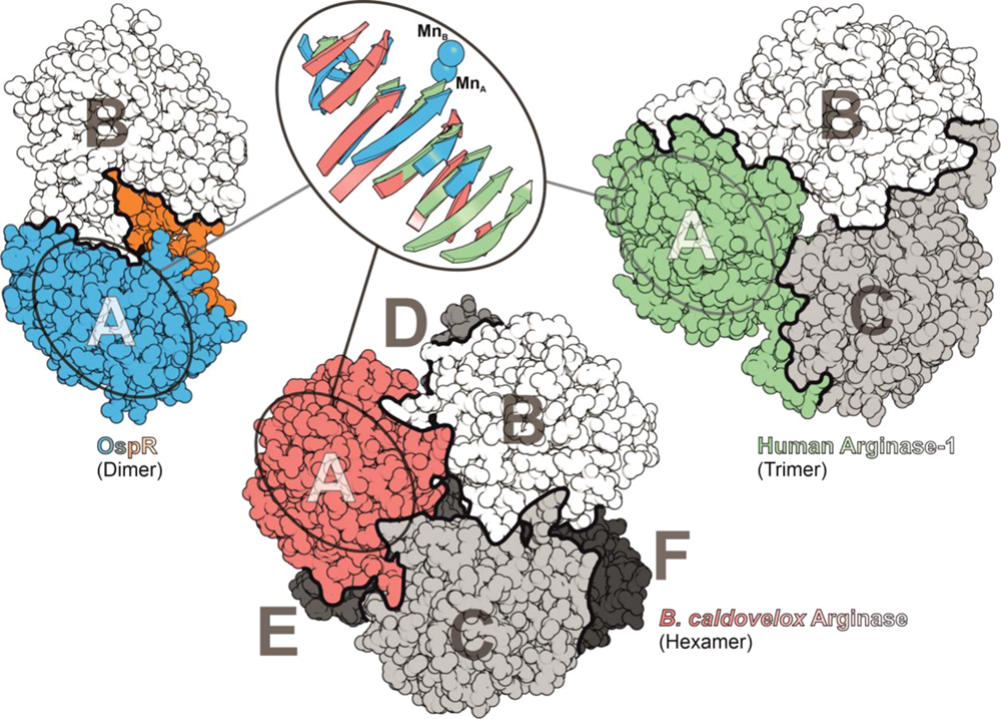
OspR displays a novel quaternary structure compared to conventional arginases. Single subunits of OspR and arginases from *B. caldovelox* (PDB-ID 2CEV) and *Homo sapiens* (PDB-ID 3GMZ) were superpositioned according to their β-sheet topology (Mn_A_ and Mn_B_ of OspR are depicted). Each oligomer is shown in similar orientation to illustrate significant differences in their quaternary assemblies (subunit A in color). Borders between subunits are highlighted by black lines. The dimerization domain of OspR is orange.

The structure of OspR reveals a three-layered α-β-α sandwich arrangement, which is a fold shared by all arginases (Figures 4A and 5).^30^ Apart from this similarity, the macromolecular assembly of OspR shows major differences compared to other arginases. As confirmed by analytical size exclusion chromatography (Figure S18), OspR forms a homodimer in solution (Figures 4A,B and 5). Additionally, PISA (Proteins, Interfaces, Structures and Assemblies) analysis^31^ of the obtained crystal structure indicated an average interface area of 1864 Å^2^ between the two subunits of the homodimer.^31^ The complex formation significance score assigned by the PISA webserver tool also supports a dimeric oligomerization state of OspR. Other protein contact areas within the crystal lattice were assigned to be the result of crystal packing rather than physiological complex formation. The dimer contact region is almost exclusively established by an additional domain (residues Ser170-Ser234), which is absent in conventional arginases (Figures 4A and 5). By contrast, eukaryotic arginases predominantly assemble as trimers, while bacterial arginases form hexamers.^8,20,22,32^ Thus, the quaternary structure of OspR differs significantly compared to known members of the arginase family (Figure 5). The narrow region occurring in the center of the OspR homodimer forms a large surface-exposed cleft (Figure 4B,C), which presumably acts as the binding site for the 10 kDa leader region of OspA. Interestingly, this architecture in OspR allows direct access to two negatively charged cavities that protrude into the central part of either subunit A or B (Figure 4C).

OspR exhibits a broad degree of substrate promiscuity, processing residues within a variety of core sequences attached to the OspA leader, as well as in distinct precursor proteins.^1^ While being flexible regarding the amino acids neighboring the targeted arginine residues, OspR does not process single molecules of arginine (Figure S7). Likewise, conventional arginases do not tolerate changes at the amino or carboxyl groups of their free arginine substrate.^33^ From a macromolecular perspective, OspR’s requirement for peptidyl substrates might be explained by its unusual oligomerization. Although we were unable to crystallize OspR in the presence of OspA to gain experimental evidence for this hypothesis, the alternative arrangement of subunits could be an elegant evolutionary adjustment to accommodate the recognition and processing of large peptide substrates while conserving the arginase reaction within the active site. The dimerization region of OspR exhibits both a high degree of flexibility, suggested by the number of random coils (Figure 4A), and a large surface-exposed cleft (Figure 4B,C), both traits suitable for binding the sizable OspA substrate. Moreover, the two active sites in the OspR homodimer are located in close proximity (Figure 4C), which could play a role in synchronizing the conversion of several Arg within the OspA core sequence or an OspA oligomer.

**Figure 6:**
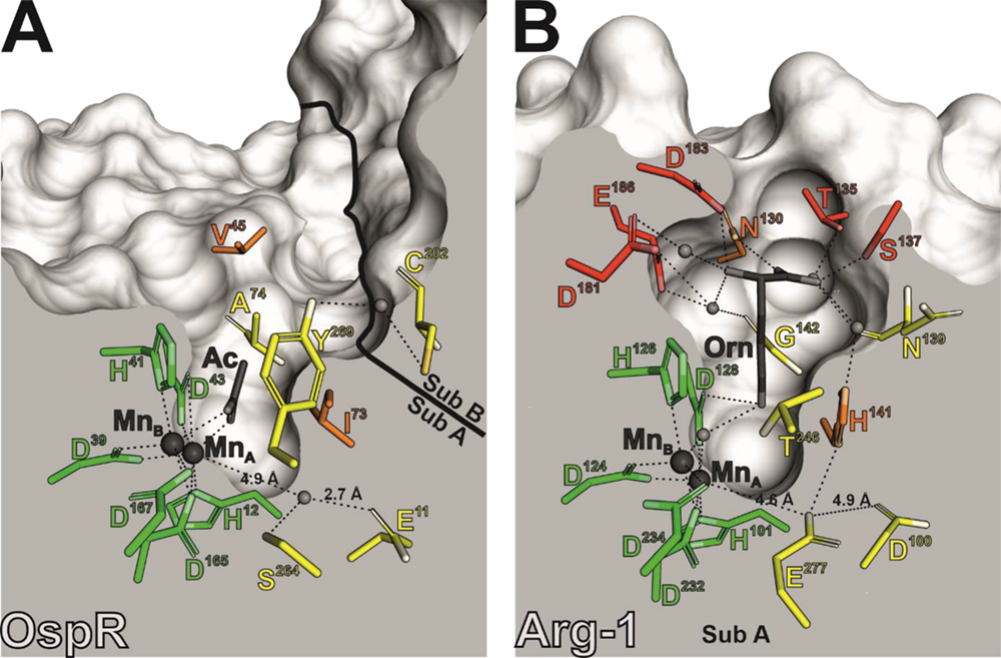
Residue conservation within the active sites of OspR (A) and subunit A of the human arginase-1 trimer (B, PDB-ID 3GMZ). Residues are represented as sticks and labeled by one-letter-codes. The color-coding indicates the degree of amino acid conservation between the two enzymes (green: conserved; yellow: similar, displaced, or protein backbone interactions; orange: not conserved; red: only existent in human arginase-1). Remaining parts of the enzymes are colored in different shades of gray. Manganese ions are shown as dark gray and selected water molecules as light gray spheres. The catalytically important Glu277 of human arginase-1 is replaced by Ser264 in OspR. We propose that its function is taken over by the neighboring Glu11, which is connected to the active site via a water molecule. Notably, almost all residues involved in binding of the carboxyl moiety and the α-amino group of ornithine in human arginase-1 are missing in OspR.

As different as OspR appears from conventional arginases on a macromolecular level, taking a closer look at the active site reveals striking similarities. The base of the negatively charged cavity (Figure 4C) accommodates a binuclear manganese cluster typical for arginases. Despite the distant sequence relationship, three binding motifs that are obligatory for complexation of such a manganese cluster are strictly conserved (Figures 1E,F, 4D,E, and S3 and S4). Mn_A_ of OspR is coordinated by His12 and Asp43, whereas Mn_B_ binds to His41 and Asp167. Asp39 and Asp165 complex both metal ions. Interestingly, the OspR structure features an acetate molecule that is bound at the substrate binding site serving as a mimic for the guanidine moiety of the actual substrate. The superposition of its catalytic center with human arginase-1 shows the extent of conservation of manganese-binding residues within peptide arginases (Figure 4D,E). This similarity suggests a high degree of accordance between the well-established reaction mechanism of conventional arginases and that of OspR, which we propose here. Catalysis starts with deprotonation of a water molecule and complexation of the resulting hydroxide ion by Mn_A_, Mn_B_, and Asp43. The hydroxide ion performs a nucleophilic attack on the carbon atom of the guanidine moiety of arginine. In the resulting tetrahedral intermediate, a proton is transferred from the added hydroxide to Asp43. During the whole reaction, the nearby Glu11 might be crucially involved in stabilization of the guanidine moiety. Upon dissociation of ornithine, urea remains in the active site in which the hydroxide ion complexed by the manganese cluster has now turned into the carbonyl moiety of urea. In the subsequent steps, urea is replaced by water, which allows the reaction cycle to restart (Figure S19).^3,7−9,11,20^ The more distant the residues are positioned from the manganese center, the less conserved they are. Glu11 is already slightly misplaced compared to human arginase-1, making room for an additional water molecule (Figure 6A). Furthermore, the established arginase mechanism features a histidine residue that serves as an important proton shuttle. This histidine is replaced by Ile73 in OspR, a residue which is unable to perform the same task. Further residues that are normally involved in complexation of the arginine backbone are also either not conserved or, for the most part, missing in OspR, as can be seen in the active-site comparison of OspR and human arginase-1 (Figure 6).^33,34^ The relatively shallow active-site pocket of OspR might eliminate the need for a proton shuttle.

Recognition of the protein substrate OspA can be accomplished by structural interactions, circumventing the need for backbone recognition of arginine. OspR binds OspA approximately three orders of magnitude more tightly (*K*_M_ 6–9 μM, Figure 2B) than conventional arginases for arginine (for example, *K*_M_ of 2.3 mM for human arginase-1),^35^ which is likely reflective of the extended protein–protein interactions of substrate recognition by peptide arginases. How the mechanism of OspR is achieved within the context of a whole peptide compared to a single arginine residue remains to be addressed. Ultimately, our findings fuel efforts to obtain molecular insights into a complex structure of a peptide arginase together with its substrate to better understand the intricacies of these fascinating enzymes.

## Conclusions

In this study, we provide a detailed biochemical and structural analysis of the atypical peptide-modifying arginase OspR and compare it to conventional arginases acting on arginine monomers. Our results illuminate key features of arginine-to-ornithine transformations in a peptide substrate and provide critical insights into the post-translational maturation steps during biosynthesis of the antiviral RiPP landornamide A. Although OspR subunits retained the universal arginase fold, its dimeric quaternary structure was unique among other characterized arginases, which generally form trimers or hexamers. Despite a low protein sequence similarity and the altered oligomeric state, binding of the binuclear manganese cluster, the active-site environment, and formation of urea as the coproduct are shared between OspR and conventional arginases. This suggests that the hydrolytic enzyme mechanism is conserved but that substantial structural adjustments were necessary to translate the catalysis of arginine-to-ornithine conversion into the context of a large peptide substrate. OspR is highly promiscuous, but it prefers the cyclized-and-epimerized OspA over linear OspA variants, clarifying that ornithines are preferentially installed as the last post-translational modification in landornamide A biosynthesis.

Most known peptide natural products are biosynthesized by two fundamentally different processes: condensation of individually selected (often nonproteinogenic) amino acids by nonribosomal peptide synthetases (NRPSs) and post-translational modification of ribosomally translated precursor proteins by RiPP pathways. Ornithines are one of many noncanonical amino acids now known to be installed in both NRPS and RiPP products. Examples of nonribosomal peptides with ornithine residues include the cyclic lipopeptides daptomycin^36^ (in clinical use) and kahalalide F,^37^ with antibiotic and anticancer activities, respectively. The antiviral peptide landornamide A,^2^ as well as the lipopeptides kamptornamide A and phaeornamide A,^38^ are the first examples of ornithine-containing RiPPs matured by pfam12640 peptide arginases. More recently, a second type of peptide arginase belonging to pfam00491 was described in the maturation of enteropeptins.^39^ In these RiPPs, ornithine formation was only possible following installation of a thiomorpholine post-translational modification at the Arg-C_α_, suggesting that the substrate tolerance for enteropeptin-type arginases is more restrictive than for OspR-type arginases. The broad substrate specificity of the characterized OspR-type peptide arginases coupled with the biochemical and structural insights provided in this study lays the foundation for further investigations into pathway engineering of landornamide variants and custom ornithine-containing peptides. As ornithines can be further enzymatically or chemically modified, arginine-to-ornithine conversions offer a basis for further structural diversification.

## Methods

Detailed methods are provided in the Supporting Information (SI).

### Cloning, Expression, and Purification

For plasmid amplification and cloning, the strain *E. coli* DH5α (Invitrogen) was used. All constructed plasmids were verified by sequencing. The cloning steps were carried out using the recommended protocols provided by the corresponding manufacturers. DNA was visualized by gel electrophoresis on 1% (w/v) agarose gels supplemented with ethidium bromide in TAE buffer. Proteins were produced in *E. coli* BL21(DE3) and purified by Ni^2+^-affinity chromatography. For details of the different expression constructs and purification conditions, see the SI.

### Enzymatic Assays

Two different types of in vitro assays were used to determine the arginase activity, a spectrophotometric assay and an LC-HRMS-based assay. For the spectrophotometric assay, the QuantiChrom Urea Assay Kit from BioAssay Systems was employed using the manufacturer’s protocol. Detailed assay conditions are described in the SI.

### HPLC-MS/MS^2^ Analysis

HPLC-MS analyses were performed on a Dionex Ultimate 3000 UHPLC coupled to a Thermo Scientific Q Exactive Hybrid Quadrupole-Orbitrap Mass Spectrometer using heated electrospray ionization in positive ion mode as described previously^1^ unless otherwise stated in the experiment-specific sections in the SI.

### ICP-MS Analysis

ICP-MS was performed by the MS service of the University of Zurich (UZH). The measurements were conducted by an inductively coupled plasma triple quadrupole mass spectrometer equipped with a standard microflow sprayer or an apex-IR nebulizer (Agilent 8800 ICP-MS).

### Crystallization Conditions

Crystals of OspR were obtained via sitting drop vapor diffusion in Intelli 96-well plates (Art Robbins Instruments). Droplets for vapor diffusion comprising 0.2 μL of protein mixed with 0.2 μL of reservoir solution were prepared against reservoir solutions of 50 μL on Intelli 96-well sitting drop plates (Art Robbins Instruments). The sealed plates were incubated at 20 °C. Crystals were identified by using a transmission microscope. Crystals of OspR grew in droplets generated from reservoir solution containing 200 mM magnesium acetate, 100 mm Tris/HCl pH 7.5, and 25% (w/v) PEG 3350. In preparation for data acquisition, crystals were cryoprotected by a 7:3 mixture of mother liquor and 100% (v/v) glycerol and subsequently vitrified in liquid nitrogen.

### Structure Determination

Datasets of OspR crystals were recorded using synchrotron radiation at the beamline X06SA, Swiss Light Source (SLS), Paul Scherrer Institute, Villigen, Switzerland. Reflection intensities were evaluated with the program package XDS and data reductions were carried out with XSCALE (Table S8).^40^ Experimental phases were obtained by single anomalous dispersion (SAD) methods using the peak absorption wavelength of selenium-derivatized OspR crystals (λ = 0.9798 Å). The program package SHELXD^41^ located 16 heavy atom sites using a dataset recorded to 2.6 Å. Subsequent SHARP-SAD-phasing^42^ and solvent flattening with the program DM^42^ resulted in an electron density map with phases at about 3.0 Å. The quality was sufficient to model secondary structure elements by polyalanine residues. With these improved phases, we unambiguously could assign the entire OspR sequence, the last missing secondary structures, loop connections, and the manganese atoms in the 2F_O_-F_C_-electron density map using the interactive three-dimensional graphic program COOT.^43^ After model building was completed, water molecules were automatically placed with ARP/wARP solvent.^44^ Restrained and TLS (Translation/Libration/Screw) refinements with REFMAC^45^ yielded superb *R*_work_ and *R*_free_ as well as root-mean-square deviation (rmsd) bond and angle values (Table S8).

## Supporting Information Available

Additional experimental details and materials used in this study include protein sequence alignments, SDS-PAGE of purified proteins, results of spectrophotometric urea assay, ICP-MS results, relative rate experiments, LC-HRMS spectra, and crystallographic data collection (PDF)

## Accession Codes

Coordinates for OspR have been deposited in the Protein Data Base under accession code 8BRP.
