# Supplementary material for: Structural and Biochemical Insights into Post-Translational Arginine-to-Ornithine Peptide Modifications by an Atypical Arginase
Source: ACS Chem Biol. 2023 Feb 15;18(3):528–36. doi: 10.1021/acschembio.2c00879 (PMC10028609; doi:10.1021/acschembio.2c00879)
Supplement: Supplementary file 1 — cb2c00879_si_001.pdf [file cb2c00879_si_001.pdf]

Supporting Information

Structural and Biochemical Insights into Post-translational Arginine-to-Ornithine Peptide Modifications by an Atypical Arginase

Silja Mordhorst,[a,b,#] Thomas Badmann,[c,#] Nina M. Bösch,[a] Brandon I. Morinaka,[a,d] Hartmut Rauch,[c] Jörn Piel,[a] Michael Groll,[c,*] Anna L. Vagstad[a,*]

[a] Institute of Microbiology, Eidgenössische Technische Hochschule (ETH) Zürich, Vladimir-Prelog-Weg 4, 8093 Zürich, Switzerland

[b] present address: Department of Pharmaceutical Biology, Pharmaceutical Institute, University of Tübingen, Auf der Morgenstelle 8, 72076 Tübingen, Germany

[c] Chair of Biochemistry, Center for Protein Assemblies, Technical University of Munich, Ernst-Otto-Fischer-Str. 8, 85748 Garching, Germany

[d] present address: Department of Pharmacy, National University of Singapore, 4 Science Drive 2, Singapore 117544, Singapore

[#] These authors contributed equally.

* Corresponding authors, E-mail: michael.groll@tum.de, avagstad@ethz.ch

Experimental Procedures

Materials

Unless stated otherwise, chemicals were purchased from Sigma Aldrich in the highest purity available. The antibiotics kanamycin sulfate, chloramphenicol, and spectinomycin dihydrochloride, and the reducing agent dithiothreitol (DTT) were purchased from AppliChem. Isopropyl β-D-1-thiogalactopyranoside (ITPG) was ordered from Biosynth, L-arginine from Fluka, and N-Acetyl-L-arginine amide hydrochloride (N-Ac-Arg-NH_2) from Carbosynth. PCR Custom primers came from Microsynth AG (Switzerland) and Phusion™ DNA polymerase from Thermo Fisher were used for polymerase chain reactions (PCR). For DNA purification, NucleoSpin™ Plasmid and NucleoSpin™ Gel and PCR Clean-up kits from Macherey-Nagel were used. Restriction enzymes and T4 DNA Ligase were purchased from New England Biolabs, and PageRuler™ Plus Prestained protein ladder (10 to 250 kDa) was ordered from Thermo Fisher. The endoproteinase GluC came from New England Biolabs. The Urea Assay Kit QuantiChrom™ (DIUR-100) was purchased from BioAssay Systems and ethylene diamine tetra acetate (EDTA, 0.5 M, pH 8.0) was ordered from Gibco. $MnCl_2$ was used from Merck, while $MgCl_2$ and $CaCl_2$ came from Fluka; all other metal chlorides were from Sigma Aldrich.

Cloning

For plasmid amplification and cloning, the strain *E. coli* DH5α (Invitrogen) was used. All plasmids constructed were verified by sequencing (Microsynth AG, Switzerland, or Eurofins Genomics, Ebersberg, Germany). The cloning steps were carried out using the recommended protocols provided by the corresponding manufacturers. DNA was visualized by gel electrophoresis on 1% (w/v) agarose gels supplemented with ethidium bromide in TAE buffer.

The cloning procedures of the full cluster pCDFDuet-Osp1,[1] the N-terminally His_6-tagged OspA-based expression plasmid pET28b-OspA (Kan^R),[2] and the untagged OspD-based construct pCDFDuet1-OspD (Sm^R)[3] have been described previously.

The *ospR* deletion mutant plasmid pCDFDuet1-6xH-v-OspAMD was obtained using standard restriction enzyme-based cloning. The insert was amplified from the pCDFDuet-Osp1 template[1] using the primer pair OspA-F-NdeI and OspD-R-NotI (Table S7). The PCR product was digested with *Nde*I and *Not*I and ligated into the respective restriction sites of plasmid pCDFDuet-1 (Spt^R) using T4 DNA ligase to give the expression plasmid pCDFDuet-6xH-OspAMD.

The plasmid containing an MBP-tagged *ospR* gene pCDF-6xH-MBP-v-OspR was also obtained using standard restriction enzyme-based cloning. The insert was amplified from the pCDFDuet-Osp1 template[1] using the primer pair OspR-F-NdeI and OspR-R-XhoI. The PCR product was digested with *Nde*I and *Xho*I and ligated into the respective restriction sites of plasmid pCDF-6xH-MBP-v (Spt^R) using T4 DNA ligase to give the expression plasmid pCDF-6xH-MBP-v-OspR. pCDF-6xH-MBP-v is a modified version of the commercial plasmid pCDFDuet-1 (Novagen) in which the region between BamHI and NdeI was replaced by the MBP solubility tag and TEVp cleavage site amplified from pMalc2v (NEB) using primers MBP-BamHI-F and TEV-NdeI-R.

<u>OspR variants</u>

The plasmid containing an MBP-tagged functional knock-out *ospR* gene pCDF-6xH-MBP-v-OspR-D39N/H41Q/D43N was obtained using standard restriction enzyme-based cloning. The

insert was amplified from the pCDFDuet-Osp1_OspR-D39N/H41Q/D43N template[1] using the primer pair OspR-F-NdeI and OspR-R-XhoI. The PCR product was digested with *Nde*I and *Xho*I and ligated into the respective restriction sites of plasmid pCDF-6xH-MBP-v (Spt^R) using T4 DNA ligase to give the expression plasmid pCDF-6xH-MBP-v-OspR-D39N/H41Q/D43N.

To obtain the construct pET28b-SUMO-OspR, the plasmid pCDF-6xH-MBP-v-OspR served as a template and restriction sites for *Bam*HI and *Pst*I were introduced during PCR using the primers OspR-F-BamHI and OspR-R-PstI (Table S7). Using restriction and ligation cloning, *ospR* was ligated into a modified pET-28(+) vector (Invitrogen, Darmstadt, Germany) containing an N-terminal His_6-SUMO protein construct [Smt3p from *Saccharomyces cerevisiae*].

The OspR variants with single point mutations in the metal-binding motif (DXHXD), pCDF-6xH-MBP-v-OspR-D43A and pCDF-6xH-MBP-v-OspR-D43N, were cloned with the primer pairs OspR-D43A_F and OspR-D43A_R (for D43A) or OspR-D43N_F and OspR-D43N_R (for D43N) using pCDF-6xH-MBP-v-OspR as a template. As described above, chemically competent DH5α cells were transformed with the PCR product after *Dpn*I digest.

Expression and Purification

For the biochemical characterization, proteins were produced in *E. coli* BL21(DE3) cells. House-made chemically competent cells were transformed with the expression constructs described above and single colonies were used to inoculate 5 to 10 mL overnight cultures (LB medium supplemented with the appropriate antibiotics) and grown at 37 °C overnight. For large-scale expressions in ultra-yield flasks, 800 mL LB medium (for precursors) or 800 mL TB medium (for arginases, 12 g/L tryptone, 24 g/L yeast extract, 4 mL/L glycerol, 17 mM KH_2PO_4, 72 mM K_2HPO_4) containing antibiotics were inoculated with over-night culture (1:100) and grown at 37 °C and 200 rpm until an OD_{600} of ~0.6 (LB medium) or ~1.5–2.0 (TB medium) was reached. The cultures were cooled down and expression was induced by addition of IPTG (final concentration 1 mM). Cultures were incubated at 16 °C and 200 rpm for 20 h.

The cells were collected by centrifugation (6700 × *g*, 4 °C, 45 min) and either frozen in liquid nitrogen and stored at –20 °C or directly re-suspended in sodium phosphate buffer (50 mM sodium phosphate pH 8.0, 300 mM NaCl, 10% glycerol, 20 mM imidazole; ~4 mL per g cell pellet) and lysed by sonication (30% amplitude, 2 min, 10 sec on/10 sec off). All steps were carried out at 4°C or on ice. The cell lysate was cleared by centrifugation and passed over a Ni^{2+}-affinity chromatography gravity column (Protino® Ni-NTA resin, Macherey-Nagel). His-tagged proteins were eluted with sodium phosphate buffer containing a high imidazole concentration (50 mM sodium phosphate pH 8.0, 300 mM NaCl, 10% glycerol, 250 mM imidazole). The eluted proteins were concentrated and desalted by Amicon 20 mL concentrators with 10 kDa (for precursors) or 50 kDa (for arginases) cut-off. The buffer was exchanged to storage buffer (50 mM TRIS pH 8.0, 300 mM NaCl, 10% glycerol). SDS-PAGE analysis with 15% acrylamide (National Diagnostics) resolving gel was performed to check the purity and the approximate size of the eluted proteins. Precision Plus Protein™ Dual Color Standards (BioRad) was used as a protein size reference. Protein concentrations were determined by Roti-Nanoquant (Carl Roth) using bovine albumin fraction V as a standard.

In preparation for crystallization, electrocompetent *E. coli* SoluBL21(DE3) were transformed with pET28b-SUMO-OspR via electroporation and, starting with a 30 mL overnight culture, were grown in Fernbach shaking flasks at 37 °C containing 3 L of TB medium (composition as described above) supplemented with 50 mg/L kanamycin. At an OD_{600} of 2.0, IPTG was added to a final concentration of 1 mM and incubation was continued overnight at 20 °C. Selenomethionine-labeled protein was expressed in the same strain using a protocol

previously described.[4] Cells were harvested by centrifugation, washed with 0.9% NaCl and re-suspended in 50 mL of 100 mM TRIS/HCl, pH 7.5, containing 500 mM NaCl, 10% glycerol, 5 mM β-mercaptoethanol, and 20 mM imidazole/HCl (Buffer A). The cells were lysed by sonication (80% amplitude, 4 min, 1 sec on/1 sec off). The resulting suspension was centrifuged at 40,000 × *g* for 20 min at 4 °C. The supernatant was applied to a 5 mL HisTrap HP column (Cytiva), which had been equilibrated with buffer A (flow rate: 5 mL/min) using an ÄKTA Pure system (Cytiva). Unbound or loosely associated proteins were removed by washing with buffer A. SUMO-OspR protein was eluted by applying a 50 mL linear gradient from buffer A to buffer B (100 mM TRIS/HCl pH 7.5, 500 mM NaCl, 10% glycerol, 5 mM β-mercaptoethanol, and 500 mM imidazole). Protein-containing fractions were combined and SUMO protease [Ulp1p from *Saccharomyces cerevisiae*] was added in a 1:100 molar ratio. The solution was dialyzed overnight at 4 °C against 20 mM TRIS/HCl, pH 7.5, containing 100 mM NaCl, 10% glycerol, and 5 mM β-mercaptoethanol and again applied to a HisTrap HP column equilibrated with buffer A. The flow through was concentrated to 2 mL and centrifuged at 20,000 × *g*. Size exclusion chromatography was performed using a HiLoad 16/600 Superdex 200 pg column (Cytiva) and an ÄKTA Pure system. Buffer C (20 mM TRIS/HCl pH 7.5, 100 mM NaCl, 10% glycerol, and 5 mM DTT) was used for equilibration and protein separation. A flow rate of 1.5 mL/min was used. Protein containing fractions were pooled and concentrated to at least 30 mg/mL using a 10k Amicon Ultra Centrifugal Filter (Merck Millipore). Protein concentrations were determined via Nanodrop (Thermo Scientific). During crystallization trials OspR was stored at 4 °C for up to 3 days. For prolonged storage, the protein was flash-frozen in liquid nitrogen and stored at –80 °C.

Size exclusion chromatography

Analytical size exclusion chromatography was performed using a 10/300 GL Superdex 200 pg Increase column (Cytiva) and an ÄKTA Pure system. All runs were performed in 20 mM TRIS/HCl pH 7.5, 100 mM NaCl, and 1 mM DTT. For each run, 100 µg of the respective protein was applied and eluted with a flow rate of 0.75 mL/min.

Crystallization conditions

Crystals of OspR were obtained via sitting drop vapor diffusion in Intelli 96-well plates (Art Robbins Instruments). Purified protein was diluted to a concentration of 15 mg/mL in buffer C supplemented with 1 mM of $MnCl_2$. Commercially available screens (Qiagen) were used to search for promising crystallization parameters. Crystallization screens were set up using the pipetting robot Phoenix (Art Robbins Instruments). Droplets for vapor diffusion comprising 0.2 µl of protein mixed with 0.2 µl reservoir solution were prepared against reservoir solutions of 50 µL on Intelli 96-well sitting-drop plates (Art Robbins Instruments). The sealed plates were incubated for several days at 20 °C. Crystals were identified by using a transmission microscope. Crystals of OspR grew in droplets generated from reservoir solution containing 200 mM magnesium acetate, 100 mM TRIS/HCl pH 7.5, 25% (*w*/*v*) PEG 3350. In preparation for data acquisition, crystals were cryoprotected by a 7:3 mixture of mother liquor and 100% (*v*/*v*) glycerol and subsequently vitrified in liquid nitrogen.

Structure determination

Datasets of OspR crystals were recorded using synchrotron radiation at the beamline X06SA, Swiss Light Source (SLS), Paul Scherrer Institute, Villigen, Switzerland. Reflection intensities were evaluated with the program package XDS and data reductions were carried out with XSCALE (Table S8).[5] Experimental phases were obtained by single anomalous dispersion

(SAD) methods using the peak absorption wavelength of selenium-derivatized OspR crystal (λ = 0.9798 Å). The program package SHELXD[6] located 16 heavy atom sites using a dataset recorded to 2.6 Å. Subsequent SHARP-SAD-phasing[7] and solvent flattening with the program DM[7] resulted in an electron density map with phases at about 3.0 Å. The quality was sufficient to model secondary structure elements by polyalanine residues. With these improved phases, we unambiguously could assign the entire OspR sequence, the last missing secondary structures, loop connections and the iron atom in the $2F_O$-F_C-electron density map using the interactive three-dimensional graphic program COOT.[8] After model building was completed, water molecules were automatically placed with ARP/wARP solvent.[9] Restrained and TLS (Translation/Libration/Screw) refinements with REFMAC[10] yielded superb R_{work} and R_{free} as well as root-mean-square deviation (rmsd) bond and angle values (Table S8). The crystal structure of OspR has been deposited in the RCSB Protein Data Bank under the accession code 8BRP.

Enzymatic assays

Two different analytic methods were used to assess *in vitro* arginase activity, a spectrophotometric assay and an LC-HRMS-based assay. For the spectrophotometric assay, the QuantiChrom™ Urea Assay Kit from BioAssay Systems was employed using the manufacturer's protocol. Specific *in vitro* reaction conditions and LC-HRMS parameters are provided in the experiment-specific sections below.

The spectrophotometric QuantiChrom™ Urea Assay Kit from BioAssay Systems requires an endpoint assay and EDTA was used to stop the enzymatic reaction (50 mM EDTA final concentration). Samples were immediately frozen in liquid nitrogen and stored at –20 °C until spectrophotometric analysis. Before the analysis, samples were centrifuged and transferred to a 96-well plate. After addition of the QuantiChrom™ reagents, the absorbance was measured with a Tecan M200 Pro (Plex) spectrophotometer at 430 nm and 25 °C after 15 sec mixing (orbital shaking) to determine the concentration of the co-product urea. All assays were performed in triplicate.

Results and Discussion

Sequence analysis of arginases

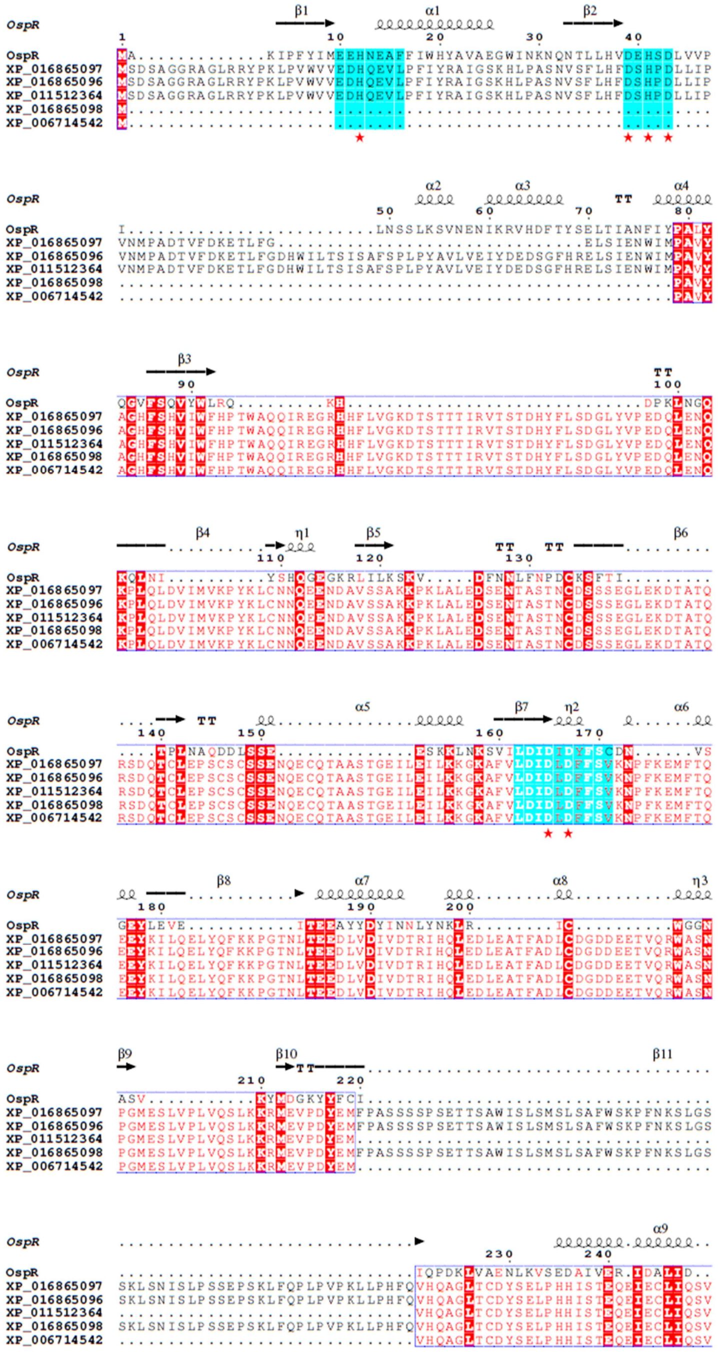

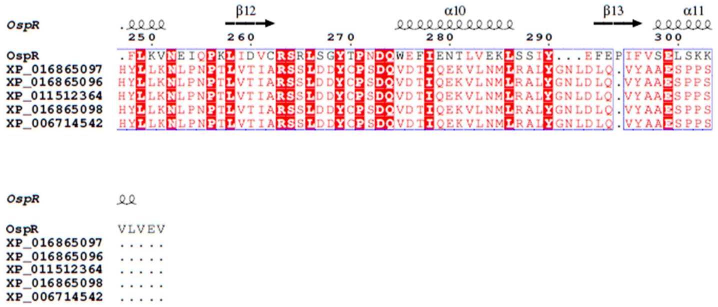

Figure S1: Multiple sequence alignment of OspR and several isoforms of human C5orf22. Protein sequences were aligned with ClustalW in MEGA11[11] using default settings. Graphics were generated in ESPript 3.0;[12] the secondary structure depiction is based on the PDB file of OspR (this work). The three sequence motifs are highlighted in cyan and residues involved in metal binding are marked with red asterisks. Abbreviations (and accession numbers): OspR (WP_007357379), XP_016865097 (UPF0489 protein C5orf22 isoform X2 [*Homo sapiens*]), XP_016865096 (UPF0489 protein C5orf22 isoform X1 [*Homo sapiens*]), XP_011512364 (UPF0489 protein C5orf22 isoform X3 [*Homo sapiens*]), XP_016865098 (UPF0489 protein C5orf22 isoform X4 [*Homo sapiens*]), XP_006714542 (UPF0489 protein C5orf22 isoform X5 [*Homo sapiens*]).

Table S1: Calculated pairwise protein sequence identities (and similarities). Abbreviations (and accession numbers): OspR (WP_007357379) and KspR (WP_007358515) from *Kamptonema sp*. PCC 6506, PhaR from *Pseudophaeobacter arcticus* (WP_027239425), ChdR from *Chitinophaga dinghuensis* (WP_111595276), CwbR from '*Candidatus* Wallbacteria bacterium' (PKK89478), BlhR from *Blautia hydrogenotrophica* (WP_005946677), PacR from *Paramaledivibacter caminithermalis* (WP_073152451), human c5orf22 isoform X3 (XP_011512364), human arginase-1 (liver enzyme, PDB 3SJT), *Bc*ARG from *Bacillus caldovelox* (PDB 1CEV), human arginase-2 (mitochondrial enzyme, PDB 1PQ3), *Rn*ARG from *Rattus norvegicus* (liver enzyme, PDB 1RLA).

**Substrate**	**Arginase**	**OspR**	**humanARG1**
**Peptidyl arginine**	**OspR**	100% (100%)	12.4% (20.9%)
	**KspR**	42.9% (62.1%)	
	**PhaR**	24.9% (46.2%)	
	**ChdR**	32.4% (49.5%)	
	**CwbR**	25.1% (41.1%)	
	**BlhR**	33.1% (53.6%)	
	**PacR**	35.8% (53.3%)	
**Unknown**	**C5orf22 isoform X3**	19.1% (32.2%)	8.4% (14.5%)
**L-Arginine**	**humanARG-1**	12.4% (20.9%)	100% (100%)
	***Bc*ARG**		39.9% (59.8%)
	**humanARG-2**		57.6% (72.1%)
	***Rn*ARG**		87.0% (93.2%)

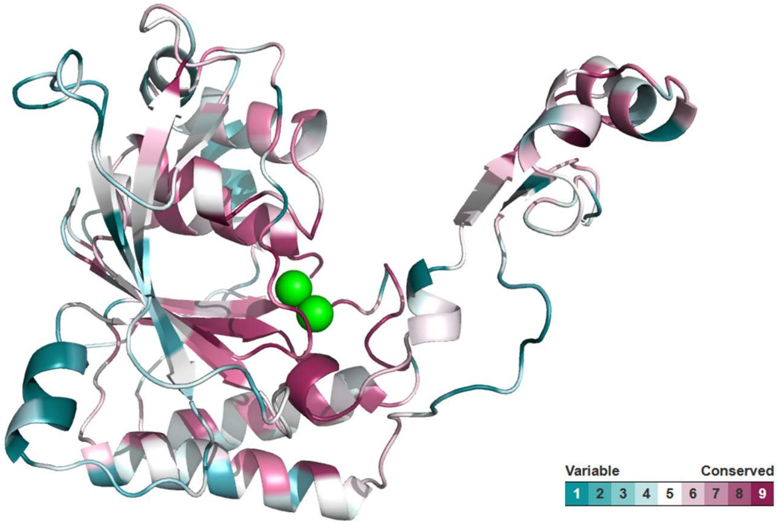

Figure S2: ConSurf[13–16] analysis shows conservation and variability among peptide arginases. The analysis was performed based on the OspR crystal structure with a multiple sequence alignment including the potential arginases identified in a previous bioinformatics analysis.[17] Conserved patches suggest structural and functional importance and are located around the active site, as expected.

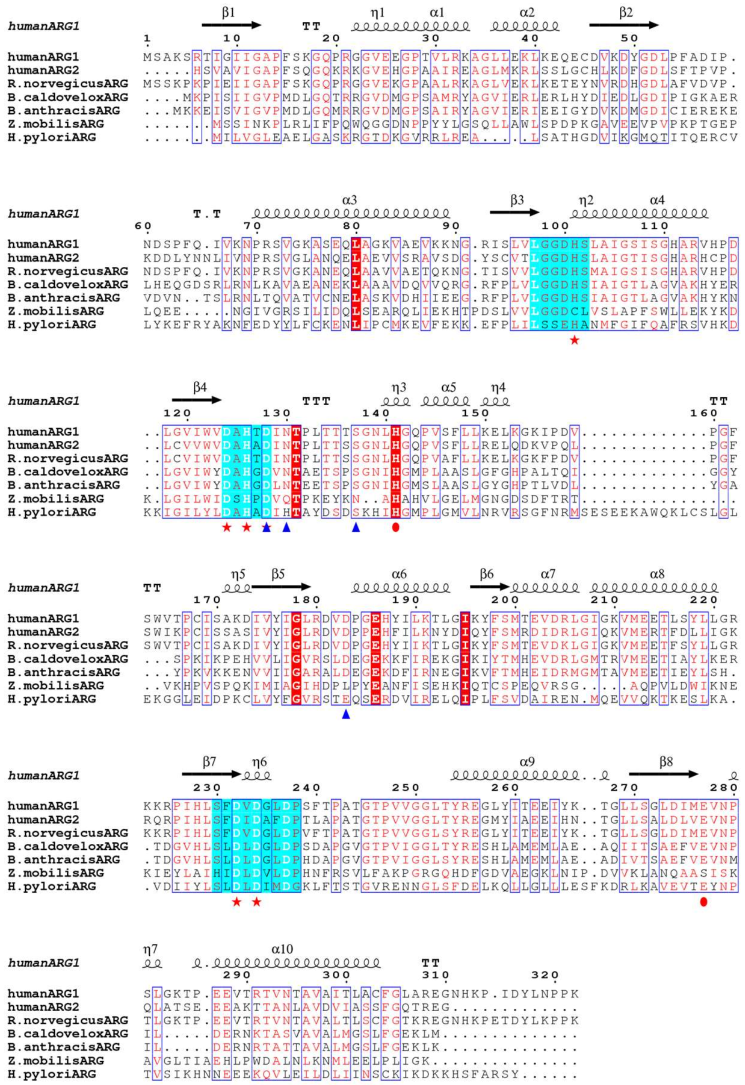

Figure S3: Multiple sequence alignment of well-characterized conventional arginases. Protein sequences were aligned with ClustalW in MEGA11[11] using default settings. Graphics were generated in ESPript 3.0;[12] the secondary structure depiction is based on the PDB file of 3SJT (human arginase-1).[18]

The three sequence motifs are highlighted in cyan, residues involved in metal binding are highlighted with red asterisks, residues involved in substrate binding with blue triangles, and residues involved in the catalytic mechanism with a red circle (Asp128 is involved in all above-mentioned actions: metal binding, substrate binding, catalysis). Abbreviations (and accession numbers): human arginase-1 (liver enzyme, PDB 3SJT), human arginase-2 (mitochondrial enzyme, PDB 1PQ3), *Rattus norvegicus* arginase (liver enzyme, PDB 1RLA), *Bacillus caldovelox* arginase (PDB 1CEV), *Bacillus anthracis* arginase (WP_000711571.1), *Zymomonas mobilis* ZM4 arginase (PDB 6KSY), *Helicobacter pylori* arginase (WP_000604403.1).

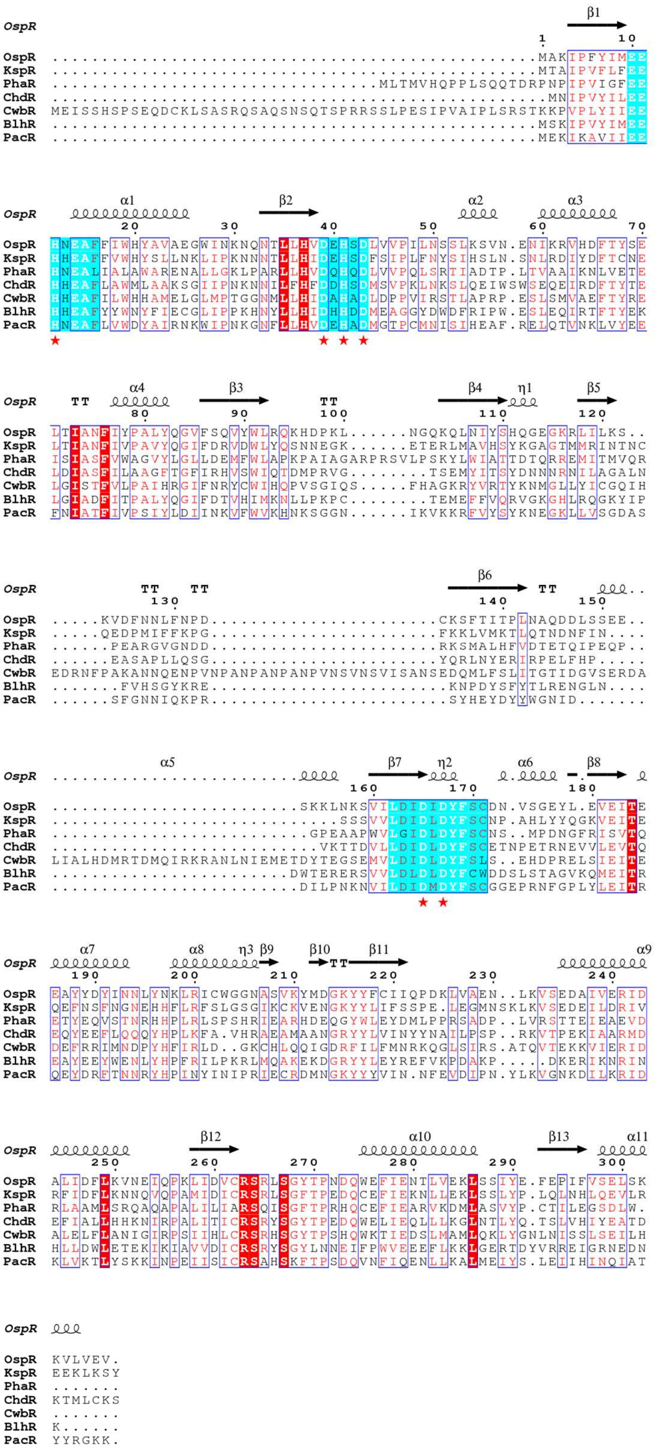

Figure S4: Multiple sequence alignment of all seven experimentally characterized peptide arginases.[17]

Protein sequences were aligned with ClustalW in MEGA11[11] using default settings. Graphics were generated in ESPript 3.0;[12] the secondary structure depiction is based on the PDB file of OspR (this work).

The three sequence motifs are highlighted in cyan and residues involved in metal binding are marked with red asterisks. Abbreviations (and accession numbers): OspR (WP_007357379) and KspR (WP_007358515) from *Kamptonema* sp. PCC 6506, PhaR from *Pseudophaeobacter arcticus* (WP_027239425), ChdR from *Chitinophaga dinghuensis* (WP_111595276), CwbR from '*Candidatus* Wallbacteria bacterium' (PKK89478), BlhR from *Blautia hydrogenotrophica* (WP_005946677), PacR from *Paramaledivibacter caminithermalis* (WP_073152451).

SDS-PAGE analysis of purified proteins

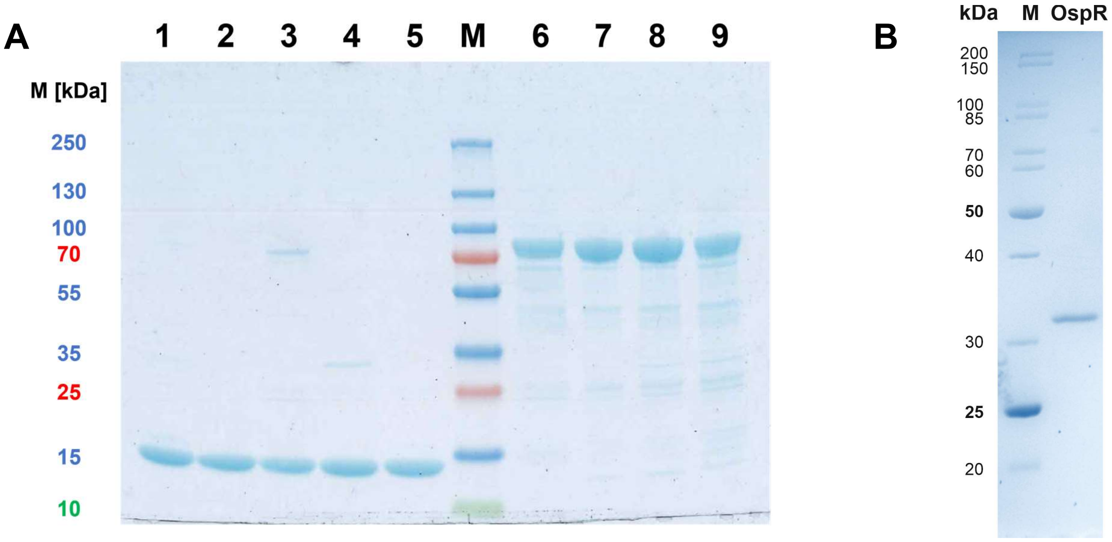

Figure S5: SDS-PAGE analysis of Ni^{2+}-affinity chromatography purified precursor proteins and peptide arginase variants. **A**) For each protein, 5 μg was loaded per well. **1** – OspA (unmodified), **2** – OspA–D-Ile4–D-Val13 ($OspA^D$), **3** – OspA–D-Ile4–D-Val13–LanCys2-Ser7–MeLanCys10-Thr14 ($OspA^{MD}$), **4** and **5** – OspA variants not reported in this study, **M** – marker, **6** – OspR-wt, **7** – OspR-Asp43Ala, **8** – OspR-Asp43Asn, **9** – OspR-Asp39Asn-His41Gln-Asp43Asn. The calculated molecular weight is ~12.8 kDa for OspA variants and ~80.4 kDa for OspR variants (including His- and MBP-tags). **B**) 1 μg SEC-purified OspR without SUMO-tag (calculated weight: 35.9 kDa).

Buffer screening for *in vitro* OspR activity assays

In a first assay, the optimal buffer conditions were identified. 20 μM OspR was incubated with 200 μM OspA (substrate) in different environments: Three different manganese concentrations (0 mM, 1 mM, and 10 mM), three different incubation temperatures (25 °C, 30 °C, and 37 °C), and three different buffer compositions (phosphate buffer, HEPES buffer, and TRIS buffer, all 50 mM and pH 8.0) were tested. Afterwards, six different pH values were tested for the TRIS buffer system ranging from 7.0 to 9.5. Samples were incubated for 4 hours and analyzed by spectrophotometric analysis (see Figure S6).

All further experiments were performed in 50 mM TRIS buffer at pH 8.5 and 1 mM $MnCl_2$. Samples were incubated at 25 °C.

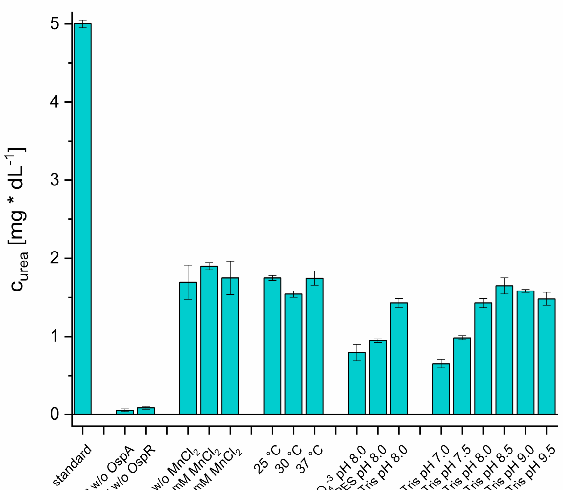

Figure S6: OspR activity under different conditions. The urea formation was determined by using the QuantiChrom™ Urea Assay Kit (see Methods section above) at 430 nm.

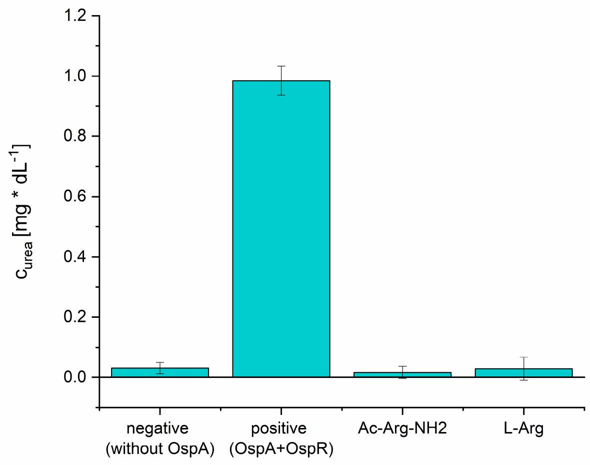

Figure S7: Activity of OspR with different substrates. OspR did not accept the single amino acid L-arginine, nor the capped arginine analogue N-Acetyl-L-arginine amide (Ac-Arg-NH_2).

Michaelis-Menten kinetics for OspR

The kinetic parameters of OspR were determined in 500 µL reaction mixtures containing 50 mM TRIS pH 8.5, 1 mM $MnCl_2$, 1 mM DTT, and 5–1000 µM substrate (OspA or OspA–D-Ile4–D-Val13–LanCys2-Ser7–MeLanCys10-Thr14). The reactions were initiated by addition of OspR at the following concentrations: 5 µM for OspA; 1 µM for OspA–D-Ile4–D-Val13–LanCys2-Ser7–MeLanCys10-Thr14. At selected time points, 50 µL aliquots were withdrawn, and the reaction was quenched by addition of EDTA. Precipitated protein was removed by centrifugation (12'044 × *g*, 5 min) and the supernatant was analyzed with QuantiChrom™ Urea Assay Kit. The time course of urea formation was fitted to a linear function to give the initial rates of reaction, which were then used to calculate specific activity and k_{cat} values. The analysis was performed with the non-linear curve fit function "MichaelisMenten" and the Levenberg Marquardt iteration algorithm of the program OriginPro (OriginLab).[19]

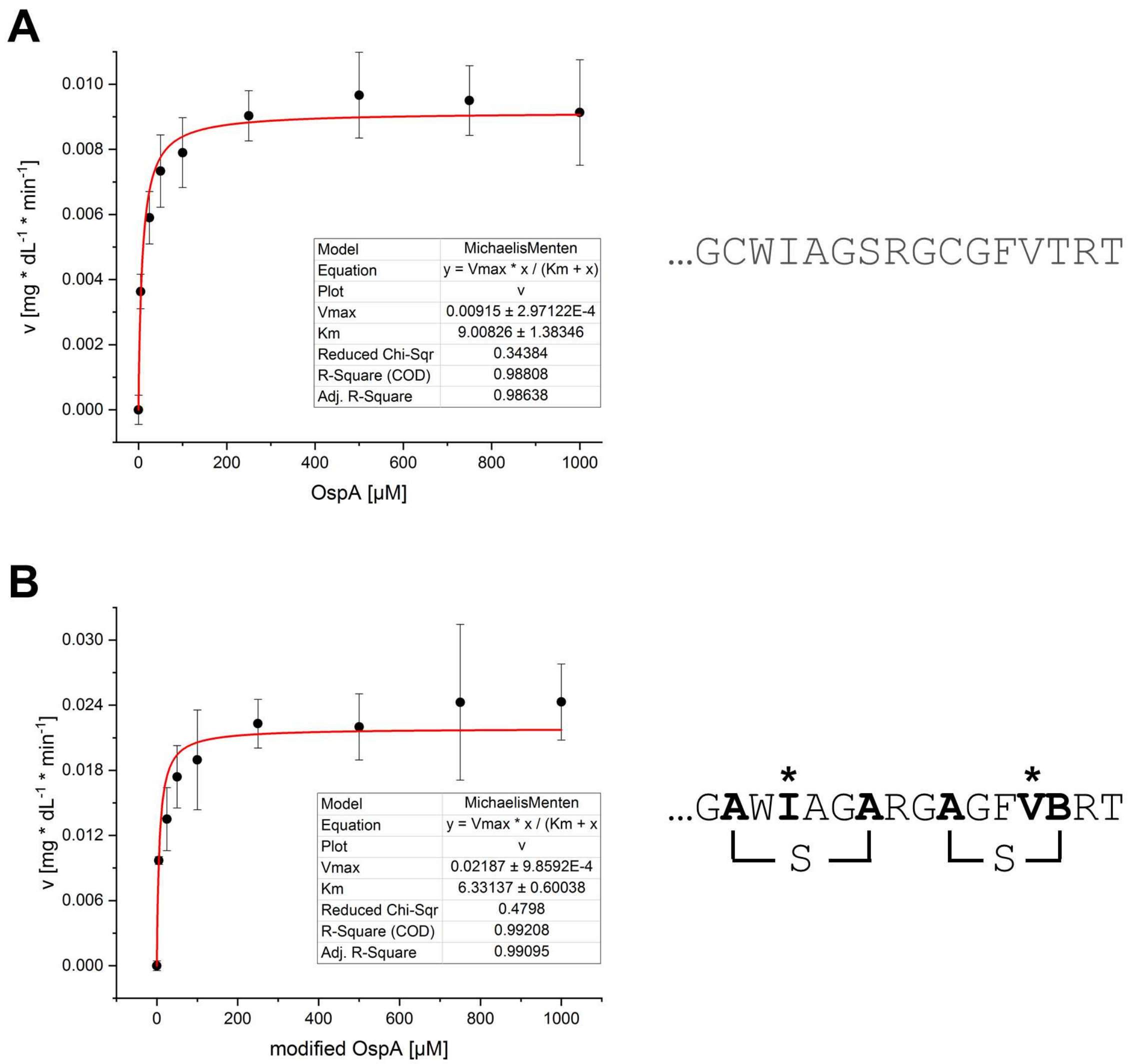

Figure S8: Michaelis-Menten kinetics of OspR with two different substrates; **A**) linear, non-epimerized OspA (unmodified), **B**) modified OspA = cyclized and epimerized OspA (OspA–D-Ile4–D-Val13–LanCys2-Ser7–MeLanCys10-Thr14). Linear substrate depiction on the right hand: the OspA core peptide $OspA_{1\text{-}16}$ is shown, asterisks highlight D-amino acids, B = butyrine.

Table S2: Kinetic data of OspR with two different substrates.

	**OspA**	**OspA–D-Ile4–D-Val13–LanCys2-Ser7–MeLanCys10-Thr14**	**Relative values (modified OspA/OspA)**
K_M	9.01 ± 1.38 µM	6.33 ± 0.60 µM	0.70
v_{max}	0.009 mg urea dL^{-1} min^{-1}	0.022 mg urea dL^{-1} min^{-1}	2.44
k_{cat}	5.078 * 10^{-3} s^{-1}	60.7 * 10^{-3} s^{-1}	11.95
k_{cat}	0.3 min^{-1}	3.6 min^{-1}	12.00
k_{cat}/K_M	564 $M^{-1}s^{-1}$	9589 $M^{-1}s^{-1}$	17.00

LC-HRMS assay

Relative rates for OspA variants differing in their epimerization and cyclisation state were analyzed by an LCMS-based assay for detection of peptide products. Three different substrates (OspA, OspA–D-I4–D-V13, and OspA–D-I4–D-V13–LanC2-S7–MeLanC10-T14) were purified by Ni^{2+}-affinity chromatography following (co)expression of OspA alone or with either OspD or OspD and OspM in *E. coli*. Reaction mixtures (100 μL) contained 50 mM TRIS pH 8.5, 1 mM $MnCl_2$, 5 μM OspR, and 150 μM OspA-variant substrate; reactions were initiated upon substrate addition. At selected time points (between 5 and 120 min), 10 μL aliquots were withdrawn and quenched with 10 μL formic acid (0.5 M), frozen in liquid nitrogen, and stored at –20 °C.

The modified substrates were digested with the endoproteinase GluC: 5 μL of the quenched reaction were neutralized with 4 μL NaOH (0.3125 M) and incubated with 0.1 μg GluC in 1x GluC buffer containing 1 mM DTT over-night at room temperature. Before analysis, the digests were supplemented with 10 μL of 1.5 mM DTT, incubated for 15 min, and diluted with 70 μL of 0.1% formic acid to a final volume of 100 μL for LCMS analysis. 5 μL were injected for each run.

For detection of the GluC-digested OspA core fragments, the following HPLC method 1 was used: column, Phenomenex Kinetex® 2.6 µm XB-C18 100 Å (50 × 4.6 mm); 1 mL/min flowrate; 50 °C column temperature; solvent A = H_2O + 0.1% formic acid, solvent B = acetonitrile + 0.1% formic acid; hold 0 to 0.2 min 85% A and 15% B, linear gradient from 0.2 to 5 min to 73% A and 27% B; wash 100% B and re-equilibrate to starting conditions. A scan range of *m*/*z* 400-1500 for the MS. Instrumentation and all other MS settings are reported in our previous study.[17] See Table X for observed retention times for relevant species. Peak areas were calculated using the ICIS peak detection algorithm in Thermo Xcaliber Qual Browser software (version 4.1.31.9) for the substrate (0 ornithines), intermediate (1 ornithine), and product (2 ornithines) peaks. Their proportional peak areas weighted by the number of ornithines were used to calculate percent conversion at each time point as previously described.[17] Relative rates of arginine-to-ornithine modifications were calculated by linear regression analysis for the 5-30 min timepoints (Linear Fit Analysis tool of the program OriginPro, OriginLab).[19]

To identify which ornithine was modified in the intermediates, select reactions were digested with GluC and trypsin proteases and separated using the following HPLC method 2: column, Phenomenex Kinetex® 2.6 µm XB-C18 100 Å (150 × 4.6 mm); 1 mL/min flowrate; 50 °C column temperature; solvent A = H_2O + 0.1% formic acid, solvent B = acetonitrile + 0.1% formic acid; hold 0 to 0.5 min 95% A and 5% B, linear gradient from 0.5 to 10.5 min to 50% A and 50% B; wash 100% B and re-equilibrate to starting conditions. A scan range of *m*/*z* 400-1200 was used for MS1 and the expected substrate, intermediate, and product ions were targeted for MS2 by parallel reaction monitoring (PRM) performed at a resolution of 35,000 (AGC target between 1e6, maximum IT 100 ms, isolation windows in the range of 2.0 *m*/*z*). Normalized collision energy (NCE) was adjusted based on the peptide and charge state to optimize fragmentation. The resulting MS data for key peptides are described in Figure S9–13 and Table S4.

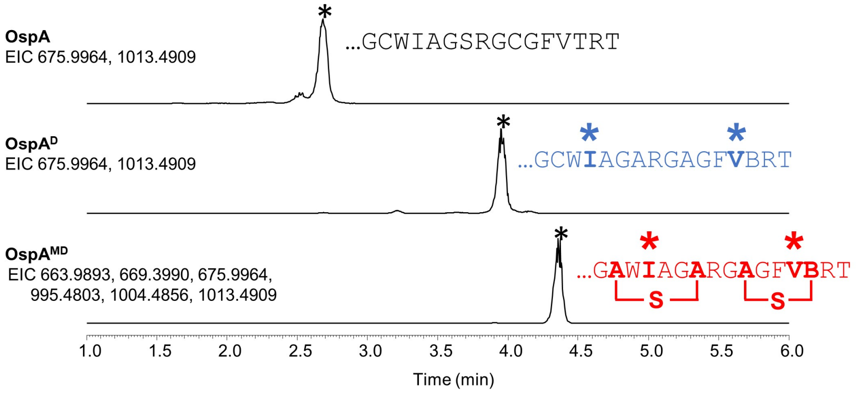

Substrate	RT (min)	Assigned structure	Expected *m/z*, *z*=3	Observed *m/z*, *z*=3	Error (ppm)
OspA	**2.68**	**unmodified**	675.9964	675.9971	1.04
	2.52	C2–C10 disulphide	675.3249	675.3245	-0.52
OspA^{D}	**3.95**	**D-*allo*-Ile4, D-Val13**	675.9964	675.9968	0.59
OspA^{MD}	**4.36**	**D-*allo*-Ile4, D-Val13, LanC2-S7, MeLanC10-T14**	663.9893	663.9900	1.05

Figure S9: LC-HRMS analysis of OspA substrates used for *in vitro* relative rate experiments. Purified precursors were digested with GluC endopeptidase. OspA$_{-5\text{-}16}$ core fragments were identified by extracted ion chromatogram (EIC) of the relevant expected $[M+3H]^{3+}$ and $[M+2H]^{2+}$ ions, as indicated. The peaks marked with an asterisk were used for relative rate calculations. RT, retention time; Lan, lanthionine; MeLan, methyllanthionine.

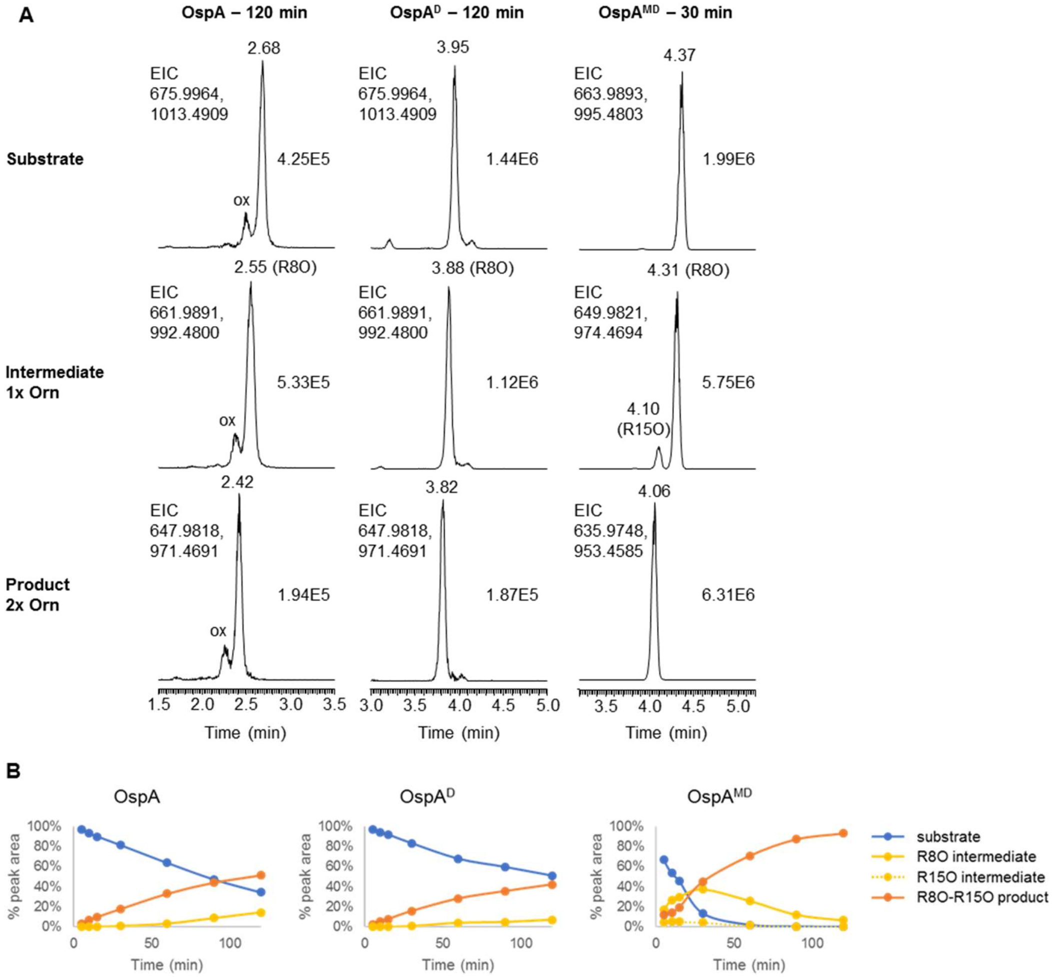

Figure S10: Representative data from OspR relative rate experiments. **A)** LC-HRMS chromatograms of a single timepoint. The OspA variant and timepoint is listed above. Substrate (top), intermediate (middle), and product (bottom) extracted ion chromatograms (EIC) for the relevant GluC-released core fragment residues –5-16, $[M+3H]^{3+}$ and $[M+2H]^{2+}$ ions ± 10 ppm are shown. The ornithine location is labelled for the single-modified intermediates as supported by trypsin digest and MS^2 data. A small amount of the oxidized (ox) Cys2-Cys10 disulfide formed spontaneously in OspA samples (left). Peak intensities are listed to the right of the individual chromatograms. **B)** Representative time course data for a single replicate from each substrate.

Table S3: Relative rates of different OspR substrates.

(Co)expression	**Substrate**	**Slope (5 to 30 min)**	**Relative rate [%]**
**OspA**	OspA (unmodified)	0.3366 ± 0.00708	24.37
**OspA^{D}**	OspA–D-*allo*-I4–D-V13	0.2935 ± 0.00689	21.25
**OspA^{MD}**	OspA–D-*allo*-I4–D-V13–LanC2-S7–MeLanC10-T14	1.38121 ± 0.0433	100.00

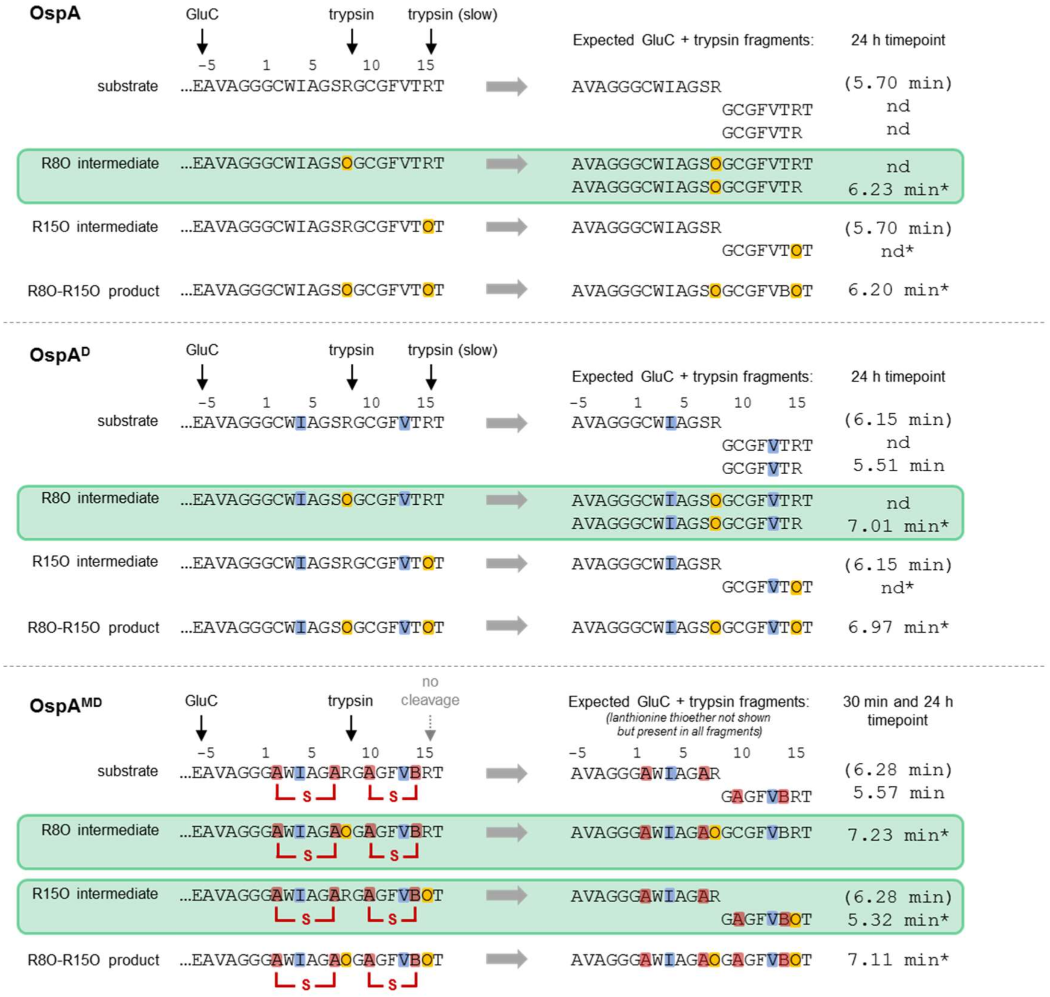

Figure S11: Single-ornithine intermediates were confirmed by digestion of relative rate reaction timepoint samples with GluC and trypsin. Ornithines are largely resistant to cleavage by trypsin leaving missed cleavage sites. Fragment LC-MS retention times are listed to the right of each fragment. Those noted with an asterisk were used to distinguish intermediates and products of the OspR reactions, and those in parentheses are shared by multiple parent species. Note that for the OspA^{MD} sample, the Arg15 site adjacent to the methyllanthionine is not cleaved by trypsin, whereas the internal Arg8 is cleaved. Key peptides were analyzed by MS^2 to confirm the location of ornithines, see Figure S12 and S13.

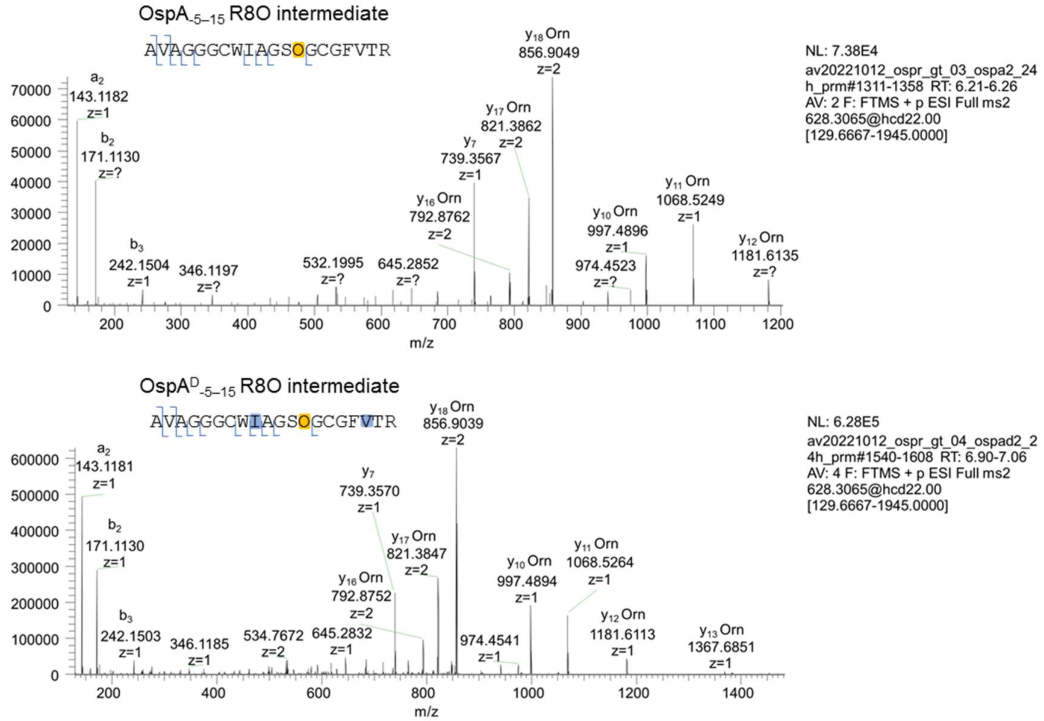

Figure S12: LC-HRMS^2 spectra of the Arg8Orn intermediate from an OspA (top) and OspA^D (bottom) substrate reaction with peptide arginase OspR (24 h timepoint). Samples were digested with GluC and trypsin. Ornithines are not appreciably cleaved by trypsin, leaving a missed cleavage site where present. The Arg15Orn intermediate was not detected for these substrates.

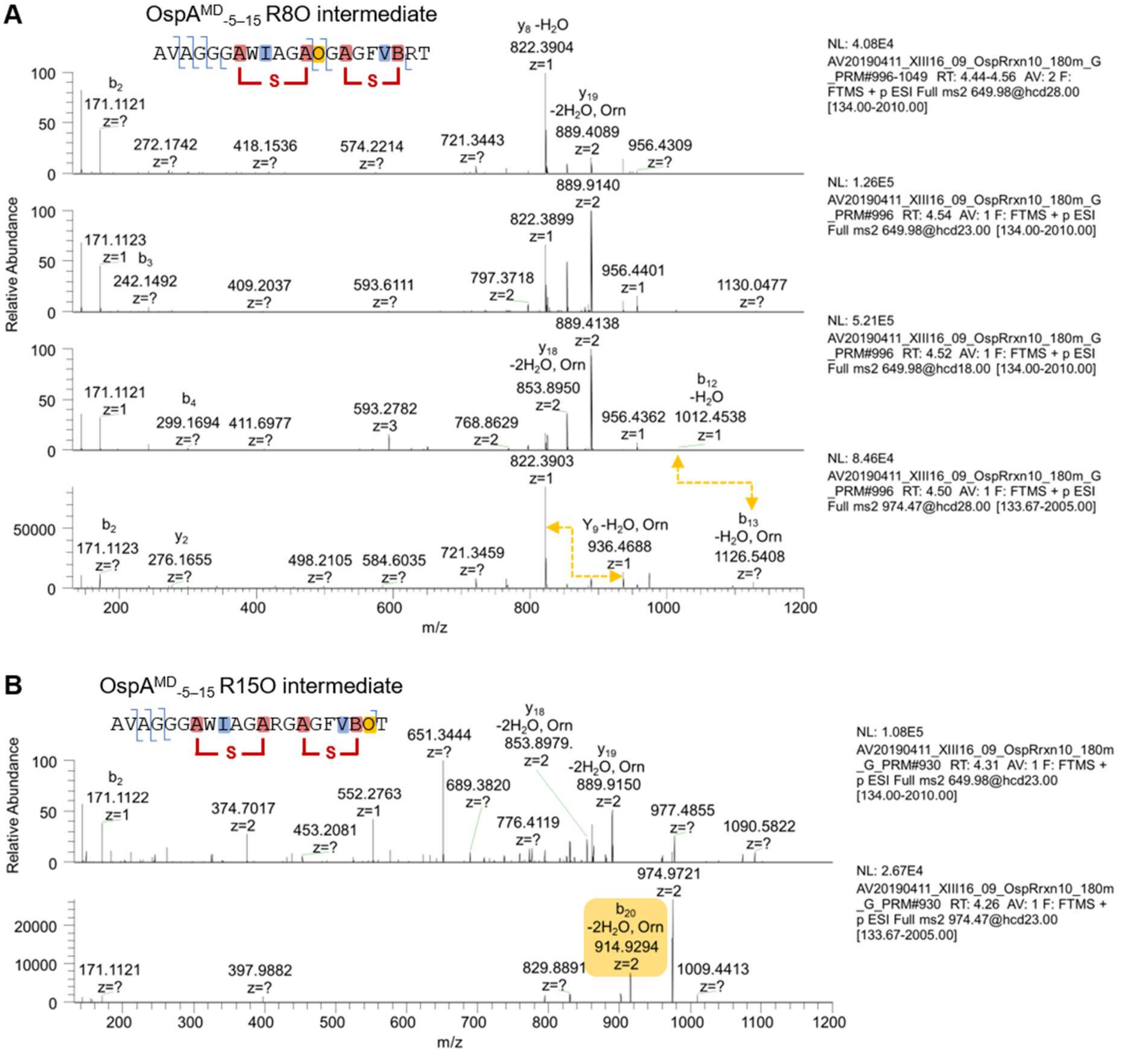

Figure S13: LC-HRMS^2 spectra of the **A**) Arg8Orn and **B**) Arg15O intermediates from an OspA^{MD} substrate reaction with peptide arginase OspR (3 h timepoint). Samples were digested with GluC. For the Arg8Orn intermediate, indicative mass shifts corresponding to installation of ornithine (Δ–42 Da) for the y8 to y9 and b12 to b13 ions were observed (yellow arrows). For the Arg15O intermediate, the b20 ion was generated, which was not visible for the Arg8Orn intermediate. The appearance of this ion is presumed to be due to the 'ornithine effect',[20] which favors fragmentation C-terminal to Orn15 due to cyclization of the sidechain onto the backbone amide ketone.

Table S4: OspA peptide fragments from GluC and GluC + trypsin digests analyzed by LC-HRMS. Retention times (RT) are provided when the species was observed. Method 1 was used for the relative rate experiment. Method 2 was used to analyze GluC + trypsin fragments to assist determination of which arginine was converted to ornithine. The expected *m/z* highlighted in blue were used for extracted ion chromatograms. All observed masses were within the 10 ppm error.

GluC fragment			Charge state:			GluC only	GluC + trypsin only
AVAGG**GCWIAGSRGCGFVTRT (-5-16)**			3	2	1	method 1	method 2
**OspA series**	M	ΔM	M+3	M+2	M+1	RT (min)	RT (min)
unmodified	2024.9673	0.0000	675.9964	1013.4909	2025.9746	2.68	nd^{T}
R8O	1982.9455	-42.0218	661.9891	992.4800	1983.9528	2.55	nd^{T}
R15O	1982.9455	-42.0218	661.9891	992.4800	1983.9528	nd	nd^{T}
R8O, R15O	1940.9237	-84.0436	647.9818	971.4691	1941.9310	2.42	6.20
**OspA^{D} series**							
D-Ile4, D-Val13	2024.9673	0.0000	675.9964	1013.4909	2025.9746	3.96	nd^{T}
D-Ile4, D-Val13, R8O	1982.9455	-42.0218	661.9891	992.4800	1983.9528	3.89	nd^{T}
D-Ile4, D-Val13, R15O	1982.9455	-42.0218	661.9891	992.4800	1983.9528	nd	nd^{T}
D-Ile4, D-Val13, R8O, R15O	1940.9237	-84.0436	647.9818	971.4691	1941.9310	3.81	6.97
**OspA^{MD} series**							
D-Ile4, D-Val13, LanC2-S7, MeLanC10-T14	1988.9461	-36.0212	663.9893	995.4803	1989.9534	4.36	nd^{T}
D-Ile4, D-Val13, LanC2-S7, MeLanC10-T14, R8O	1946.9243	-78.0430	649.9820	974.4694	1947.9316	4.30	7.23
D-Ile4, D-Val13, LanC2-S7, MeLanC10-T14, R15O	1946.9243	-78.0430	649.9820	974.4694	1947.9316	4.11	nd^{T}
D-Ile4, D-Val13, LanC2-S7, MeLanC10-T14, R8O, R15O	1904.9025	-120.0648	635.9748	953.4585	1905.9098	4.26	7.11

			Charge state:				GluC + trypsin only
**GluC + trypsin fragments**			3	2	1		method 2
	M	ΔM	M+3	M+2	M+1		RT (min)
AVAGG**GCWIAGSRGCGFVTR (-5-15)**							
unmodified	1923.9196	0.0000	642.3138	962.9671	1924.9269		nd^{T}
R8O	1881.8978	-42.0218	628.3065	941.9562	1882.9051		6.23
R15O	1881.8978	-42.0218	628.3065	941.9562	1882.9051		nd^{T}
R8O R15O	1839.8760	-84.0436	614.2993	920.9453	1840.8833		(5.22)
D-Ile4, D-Val13	1923.9196	0.0000	642.3138	962.9671	1924.9269		nd^{T}
D-Ile4, D-Val13, R8O	1881.8978	-42.0218	628.3065	941.9562	1882.9051		7.01
D-Ile4, D-Val13, R15O	1881.8978	-42.0218	628.3065	941.9562	1882.9051		nd^{T}
D-Ile4, D-Val13, R8O, R15O	1839.8760	-84.0436	614.2993	920.9453	1840.8833		(5.88)
D-Ile4, D-Val13, LanC2-S7, MeLanC10-T14	1887.8984	-36.0212	630.3067	944.9565	1888.9057		nd*
D-Ile4, D-Val13, LanC2-S7, MeLanC10-T14, R8O	1845.8766	-78.0430	616.2995	923.9456	1846.8839		nd*
D-Ile4, D-Val13, LanC2-S7, MeLanC10-T14, R15O	1845.8766	-78.0430	616.2995	923.9456	1846.8839		nd*
D-Ile4, D-Val13, LanC2-S7, MeLanC10-T14, R8O, R15O	1803.8548	-120.0648	602.2922	902.9347	1804.8621		nd*
AVAGG**GCWIAGSR (-5-8)**							
unmodified	1203.5819	0.0000	402.2012	602.7982	1204.5892		5.70
R8O	1161.5601	-42.0218	388.1940	581.7873	1162.5674		(5.60)
D-Ile4	1203.5819	0.0000	402.2012	602.7982	1204.5892		6.15
D-Ile4, R8O	1161.5601	-42.0218	388.1940	581.7873	1162.5674		(6.09)
D-Ile4,LanC2-S7	1185.5713	-18.0106	396.1977	593.7929	1186.5786		6.28
D-Ile4, LanC2-S7, R8O	1143.5495	-60.0324	382.1904	572.7820	1144.5568		nd*
**GCGFVTRT (9-16)**							
unmodified	839.3960	0.0000	280.8059	420.7053	840.4033		6.55
R15O	797.3742	-42.0218	266.7987	399.6944	798.3815		nd
D-Val13	839.3960	0.0000	280.8059	420.7053	840.4033		6.53
D-Val13, R15O	797.3742	-42.0218	266.7987	399.6944	798.3815		(5.46)
D-Val13, MeLanC10-T14	821.3854	-18.0106	274.8024	411.7000	822.3927		5.57
D-Val13, MeLanC10-T14, R15O	779.3636	-60.0324	260.7951	390.6891	780.3709		5.32
**GCGFVTR (9-15)**							
unmodified	738.3483	0.0000	247.1234	370.1814	739.3556		nd
R15O	696.3265	-42.0218	233.1161	349.1705	697.3338		nd*
D-Val13	738.3483	0.0000	247.1234	370.1814	739.3556		5.51
D-Val13, R15O	696.3265	-42.0218	233.1161	349.1705	697.3338		nd*
D-Val13, MeLanC10-T14	720.3377	-18.0106	241.1198	361.1761	721.3450		nd*
D-Val13, MeLanC10-T14, R15O	678.3159	-60.0324	227.1126	340.1652	679.3232		nd*

nd = not detected - peptide not favored due to trypsin specificity; nd^{T} = not detected - peptide cleaved by trypsin; RT in parentheses resulted from minor cleavage at Orn8*

OspR variants

The activity of the three OspR variants was determined with the spectrophotometric assay. Reaction mixtures contained 50 mM TRIS pH 8.5, 1 mM $MnCl_2$, 1 mM DTT, and 100 μM OspA. The reactions were initiated by addition of OspR (5 μM). After 4 hours, 50 μL aliquots were withdrawn, and the reaction was stopped by addition of EDTA. Precipitated protein was removed by centrifugation (12'044 × *g*, 5 min) and the supernatant was analyzed with QuantiChrom™ Urea Assay Kit.

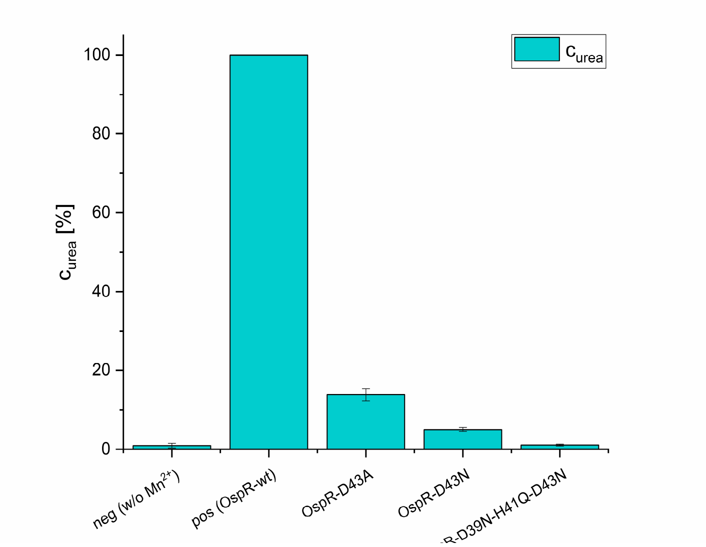

Figure S14: Activity assay of OspR variants. Urea formation was determined with the QuantiChrom™ Urea Assay Kit. Samples were incubated for 4 hours at room temperature.

Inductively Coupled Plasma Mass Spectrometry (ICP-MS) Analysis

Differently treated arginase samples were analyzed: i) OspR as purified and ii) OspR negative (treated with EDTA and dialyzed as described in section 'metal complementation assay', above). The precursor peptide OspA (as purified) was used as an additional negative control. Samples were concentrated to 1 mg/mL, then 5 mL samples were acidified with 0.167 mL HNO_3 60% ultrapure and diluted (as indicated in the sample name in Table S5) with 2% HNO_3. 3.9 mL of each sample was used for the measurement.

Table S5: ICP-MS results.
The first rows (in gray) show the results of the calibration standards (Figure S15). OspR-KO = OspR-Asp39Asn-His41Gln-Asp43Asn; CPS = counts per second, RDS = relative standard deviation.

		55 -> 55 Mn [He]			115 -> 115 In (ISTD) [He]	
**Type**	**Sample Name**	**Conc. [ng/ml]**	**CPS**	**Conc. RSD**	**CPS**	**CPS RSD**
CalBlk	0 ng/mL	0.00	200	N/A	1425757	5.50
CalStd	1 ng/mL	0.96	11104	0.20	1641613	2.30
CalStd	5 ng/mL	5.03	54736	1.00	1591974	1.10
CalStd	10 ng/mL	9.97	111661	1.80	1639371	0.60
CalStd	25 ng/mL	24.94	277869	0.10	1637386	2.60
CalStd	50 ng/mL	50.08	514086	0.20	1488455	2.10
CalStd	100 ng/mL	99.98	1110681	0.10	1619064	1.30
Sample	OspA_negative control	0.99	10218	3.20	1480819	0.30
Sample	OspR_as purified 1:10	24.24	275443	0.20	1666900	0.80
Sample	OspR_negative	28.34	287529	0.70	1488943	0.30
Sample	OspR-KO_as purified	4.26	39886	1.50	1368567	0.80
Sample	OspR-KO_negative	1.45	13076	4.50	1303438	0.70

Table S6: Calculated manganese concentrations and ratio of Mn^{2+} for the analyzed samples relative to as-purified OspR as determined by ICP-MS. OspR-KO = OspR-Asp39Asn-His41Gln-Asp43Asn mutant.

	**Mn^{2+}**		**ratio**
Sample Name	**ng/mL**	**%**	**Mn^{2+}:OspR**
OspA_negative control	0.99	0.41	0.00
OspR_as purified	242.44	100.00	0.36
OspR_negative	28.34	11.69	0.04
OspR-KO_as purified	4.26	1.76	0.01
OspR-KO_negative	1.45	0.60	0.00

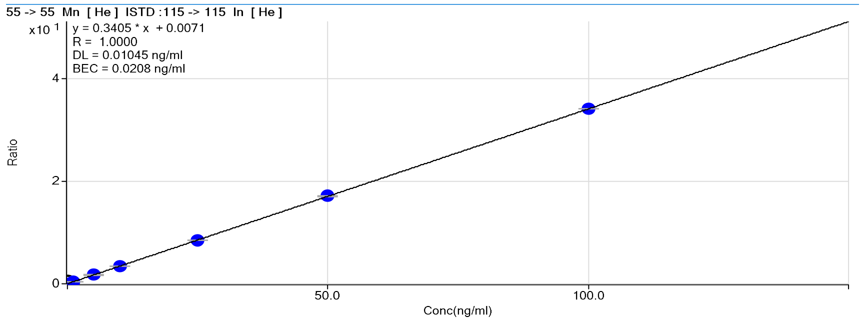

Figure S15: Calibration curve for Mn^{2+} by ICP-MS measurements.

The ratio of Mn^{2+} to OspR is expected to be 2.0 based on the crystal structure. The low occupancy of Mn^{2+} ions calculated from the ICP-MS measurements could indicate that the active site of OspR produced in *E. coli* is not fully occupied by a binuclear metal cluster. In the Mn^{2+} supplement experiment summarized in Figure S4, the as-purified OspR is fully active compared to reactions supplemented with an additional 1 or 10 mM $MnCl_2$, suggesting that the protein is at full metal-occupancy and cannot be further supplemented. Thus, the ratio at 0.36 for the as-purified OspR sample is likely lower than the expected two Mn^{2+} ions per OspR due to the sample preparation procedure for ICP-MS analysis. The measurement requires sample acidification, which led to protein precipitation in the case of OspR. It is possible, that some manganese ions co-precipitated with the protein. Generally, Mn^{2+} was added to the activity-assay buffer to ensure saturation of the OspR metal-binding sites.

Metal complementation assay

In order to analyze if other metal ions can replace manganese during catalysis, six different metal ions were tested: Mg^{2+}, Ca^{2+}, Cu^{2+}, Ni^{2+}, Co^{2+}, and Zn^{2+}.

To eliminate Mn^{2+} from the enzyme, the proteins were incubated with EDTA (1 mg protein + 100 μmol EDTA) for 3 hours at 20 °C. Afterwards, the samples were submitted to dialysis (10 kDa cut-off) and dialyzed against 1 L of protein storage buffer (50 mM TRIS-HCl pH 8.0, 300 mM NaCl, 10% glycerol) at 4 °C. After 2 hours, the buffer was exchanged, and dialysis was continued overnight. The EDTA-treated OspR-wt was immediately aliquoted and supplemented with the different metal ions (Mn^{2+}, Mg^{2+}, Ca^{2+}, Cu^{2+}, Ni^{2+}, Co^{2+}, Zn^{2+}) to stabilize the protein. Samples were incubated for 30 min on ice, flash frozen, and stored at –20 °C.

Reaction mixtures (350 μL) contained 50 mM TRIS pH 8.5, 1 mM XCl_2 (X = Mn^{2+}, Mg^{2+}, Ca^{2+}, Cu^{2+}, Ni^{2+}, Co^{2+}, Zn^{2+}), and 150 μM of the substrate OspA–D-Ile4–D-Val13–LanCys2-Ser7–MeLanCys10-Thr14. The cyclized-epimerized OspA was used as substrate for this set of reactions due to its faster rate of conversion compared to unmodified OspA. Reactions were initiated by addition of OspR (5 μM, premixed with the corresponding metal ion, see above). OspR premixed with Mn^{2+} served as a positive control, and OspR treated with EDTA and dialyzed was used as a negative control. The samples were analyzed with QuantiChrom™ Urea Assay Kit.

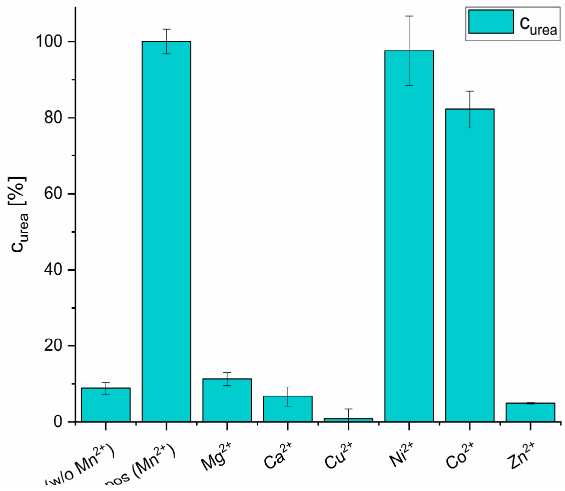

Figure S16: Metal complementation assay. Urea formation was determined with the QuantiChrom™ Urea Assay Kit. Samples were incubated for 4 hours at room temperature.

In vitro activity of untagged (SUMO-cleaved) OspR used for x-ray crystallography

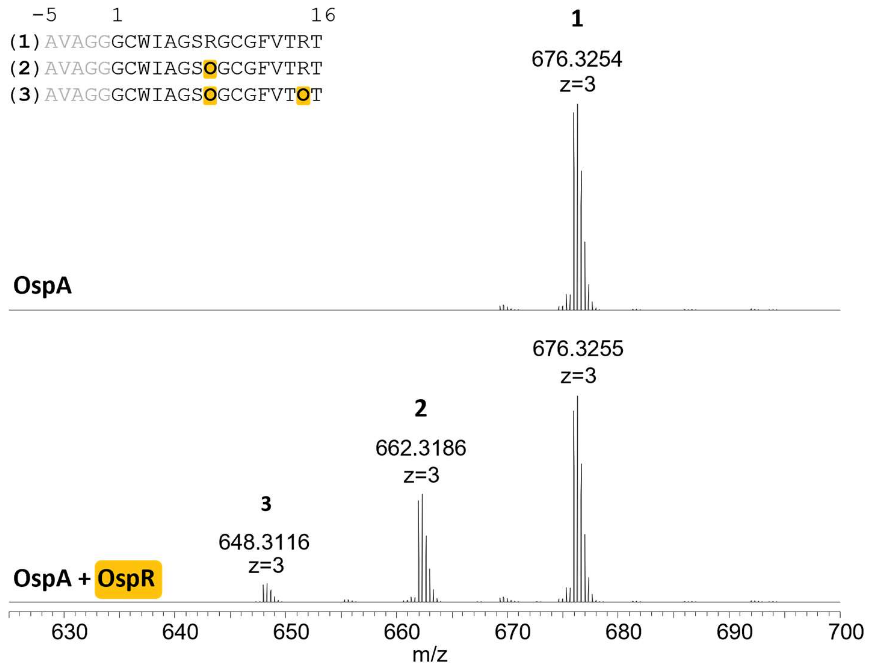

Figure S17: Untagged OspR used for x-ray crystallography is catalytically competent. *In vitro* activity of SUMO-cleaved OspR (5 μM) toward unmodified OspA (100 μM) (bottom) relative to a control lacking OspR (top) in 50 mM TRIS pH 8.5 supplemented with 1 mM $MnCl_2$. The reaction mixtures (2.5 h timepoints) were digested with GluC to release the core peptides, which were detected by LC-HRMS.

Determination of the oligomerization state of OspR by size exclusion chromatography

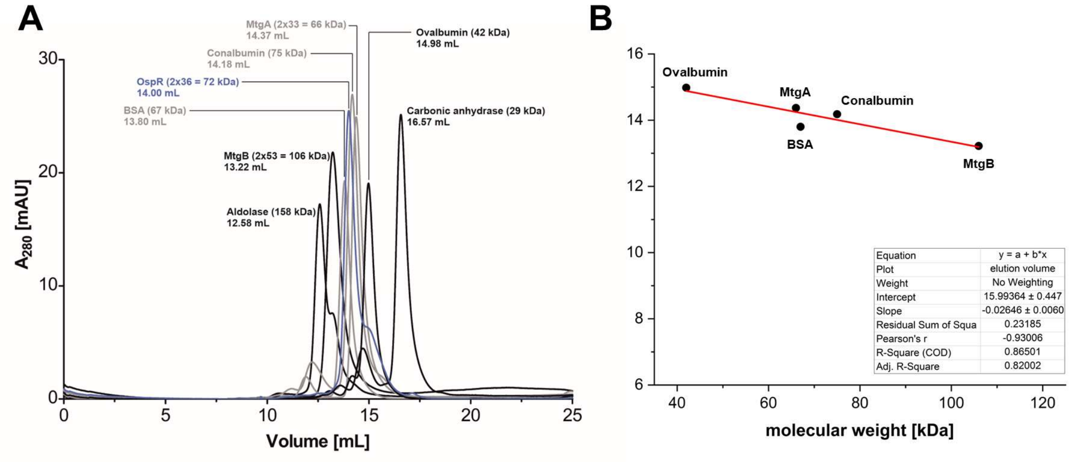

Figure S18: OspR forms a homodimer in solution. **A**) Analytical size exclusion chromatography (SEC) of untagged OspR (35.8 kDa) and molecular weight standards. The elution time of OspR (blue curve) was compared to proteins of defined sizes. The elution volume of OspR is similar to several other proteins of around 70 kDa (BSA, conalbumin, and MtgA; grey curves), suggesting a homodimeric oligomerization state (expected 71.7 kDa). Since 106 kDa MtgB elutes much earlier, trimeric or higher assemblies of OspR can be ruled out. Similarly, OspR is unlikely to form a monomer taking into consideration the late elution times of ovalbumin (42 kDa) and carbonic anhydrase (29 kDa). Note that SEC elution times are dependent on protein size and, to a small degree, shape, which might explain the irregular elution order of BSA, OspR, and Conalbumin. Protein standards were purchased from SERVA (BSA and ovalbumin) and Cytiva (aldolase, conalbumin, and carbonic anhydrase). MtgB and MtgA proteins were produced in house.[21,22] **B**) Calibration curve for size exclusion chromatography standards. OspR has a calculated molecular weight of 75.4 kDa, which closely aligns to the expected molecular weight of a homodimer (71.7 kDa).

Mechanistic proposal for peptidyl arginine hydrolysis

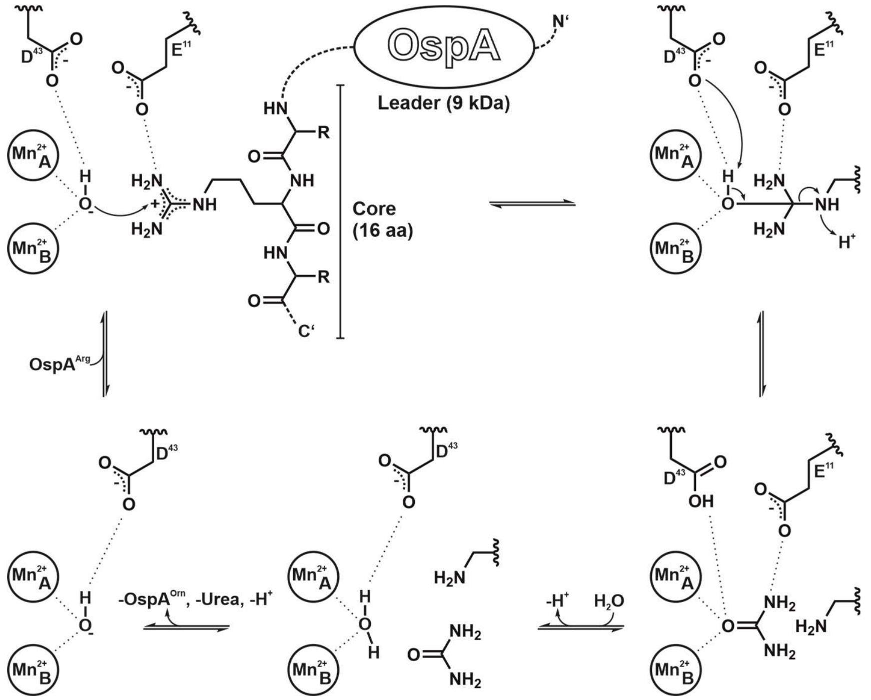

Figure S19: Proposed catalytic mechanism of OspR-catalyzed peptidyl arginine-to-ornithine hydrolysis.
The reaction cycle starts with deprotonation of a water molecule and complexation of the resulting hydroxide ion by Mn_A, Mn_B, and Asp43. The hydroxide ion performs a nucleophilic attack on the carbon atom of the guanidine moiety of the peptidyl arginine. In the resulting tetrahedral intermediate state, a proton is transferred from the added hydroxide to Asp43. During the whole reaction, the nearby Glu11 might be crucially involved in stabilization of the guanidine moiety. Upon dissociation of Orn, urea remains in the active site. The hydroxide ion complexed by the manganese cluster before has turned into the carbonyl moiety of urea. In the following steps, urea is replaced by water, which allows the reaction cycle to restart.

Table S7. Primer list.

Name	Sequence 5' → 3'
OspA-F-NdeI	GTT TTC ATA TGT CTA CTC GCA AAG AAG CCG
OspD-R-NotI	GTA AGC GGC CGC CTA CCC AGG CTG AGG TTC GTC
OspR-F-NdeI	GTA ACA TAT GGC TAA GAT TCC GTT TTA TAT CAT G
OspR-R-XhoI	GTA TCT CGA GCT AGA CCT CTA CTA AAA CTT
TEV-NdeI-R	GTA TCA TAT GTG ACT GGA AGT ACA GTC CC
MBP-BamHI-F	GAG GAT CCG AAA ATC GAA GAA GGT AAA CTG G
OspR-F-BamHI	CCA GGA TCC GCT AAG ATT CCG TTT TAT ATC ATG G
OspR-R-PstI	CCA CTG CAG TTA GAC CTC TAC TAA AAC TTT TTT AGA C
OspR-D43A_F	CTA CTT CAT GTA GAT GAA CAT TCA GCG CTA GTA GTT CCA
OspR-D43A_R	TGG AAC TAC TAG CGC TGA ATG TTC ATC TAC ATG AAG TAG
OspR-D43N_F	CTA CTT CAT GTA GAT GAA CAT TCA AAC CTA GTA GTT CCA
OspR-D43N_R	TGG AAC TAC TAG GTT TGA ATG TTC ATC TAC ATG AAG TAG

Table S8: Crystallographic data collection and refinement statistics.

	**OspR**
**Crystal parameters**	
Space group	$P2_12_12_1$
Cell constants	a = 58.3 Å<br>b = 154.4 Å<br>c = 174.9 Å
OspR / AU[b]	4
**Data collection**	
Beam line	X06SA, SLS
Wavelength (Å)	0.98
Resolution range (Å)[c]	50 – 2.6<br>(2.7 – 2.6)
No. observations	296,507
No. unique reflections	49,464[d]
Completeness (%)[c]	99.8 (99.6)
R_{merge} (%)[ce]	8.9 (60.1)
I/σ (I)[c]	15.2 (3.3)
**Refinement (REFMAC5)**	
Resolution range (Å)	30 – 2.6
No. refl. working set	46,960
No. refl. test set	2,472
No. non hydrogen	10,114
No. of manganese atoms	8
No. of solvent molecules	85
R_{work}/R_{free} (%)[f]	19.6/23.3
r.m.s.d. bond (Å) / angle (°)[g]	0.002/1.2
Average B-factor ($Å^2$)	53.6
Ramachandran Plot (%)[h]	98.8/1.1/0.1
PDB accession code	8BRP

[a] Datasets were recorded from one crystal
[b] Asymmetric unit
[c] The values in parentheses for resolution range, completeness, R_{merge} and I/σ (I) correspond to the highest resolution shell
[d] Data reduction was carried out with XDS and from a single crystal. Friedel pairs were treated as identical reflections
[e] $R_{merge}(I) = \Sigma_{hkl}\Sigma_j \mid I(hkl)_j - \langle I(hkl)\rangle \mid / \Sigma_{hkl} \Sigma_j I(hkl)_j$, where $I(hkl)_j$ is the jth measurement of the intensity of reflection hkl and <I(hkl)> is the average intensity
[f] $R = \Sigma_{hkl} \mid |F_{obs}| - |F_{calc}| \mid / \Sigma_{hkl} |Fobs|$, where R_{free} is calculated without a sigma cut off for a randomly chosen 5% of reflections, which were not used for structure refinement, and R_{work} is calculated for the remaining reflections
[g] Deviations from ideal bond lengths/angles
[h] Percentage of residues in favored region / allowed region / outlier region

References Supporting Information

(1) Bösch, N. M.; Borsa, M.; Greczmiel, U.; Morinaka, B. I.; Gugger, M.; Oxenius, A.; Vagstad, A. L.; Piel, J. Landornamides: Antiviral Ornithine-Containing Ribosomal Peptides Discovered through Genome Mining. *Angew. Chem. Int. Ed.* **2020**, *59* (29), 11763–11768. https://doi.org/10.1002/anie.201916321.

(2) Morinaka, B. I.; Vagstad, A. L.; Helf, M. J.; Gugger, M.; Kegler, C.; Freeman, M. F.; Bode, H. B.; Piel, J. Radical S-Adenosyl Methionine Epimerases: Regioselective Introduction of Diverse D-Amino Acid Patterns into Peptide Natural Products. *Angew. Chem. Int. Ed.* **2014**, *53* (32), 8503–8507. https://doi.org/10.1002/anie.201400478.

(3) Morinaka, B. I.; Verest, M.; Freeman, M. F.; Gugger, M.; Piel, J. An Orthogonal D2O-Based Induction System That Provides Insights into D-Amino Acid Pattern Formation by Radical S-Adenosylmethionine Peptide Epimerases. *Angew. Chem. Int. Ed.* **2017**, *56* (3), 762–766. https://doi.org/10.1002/anie.201609469.

(4) Van Duyne, G. D.; Standaert, R. F.; Karplus, P. A.; Schreiber, S. L.; Clardy, J. Atomic Structures of the Human Immunophilin FKBP-12 Complexes with FK506 and Rapamycin. *J. Mol. Biol.* **1993**, *229* (1), 105–124. https://doi.org/10.1006/jmbi.1993.1012.

(5) Kabsch, W. XDS. *Acta Crystallogr. D Biol. Crystallogr.* **2010**, *66* (Pt 2), 125–132. https://doi.org/10.1107/S0907444909047337.

(6) Sheldrick, G. M. A Short History of SHELX. *Acta Crystallogr. A* **2008**, *64* (Pt 1), 112–122. https://doi.org/10.1107/S0108767307043930.

(7) Bricogne, G.; Vonrhein, C.; Flensburg, C.; Schiltz, M.; Paciorek, W. Generation, Representation and Flow of Phase Information in Structure Determination: Recent Developments in and around SHARP 2.0. *Acta Crystallogr. D Biol. Crystallogr.* **2003**, *59* (Pt 11), 2023–2030. https://doi.org/10.1107/s0907444903017694.

(8) Emsley, P.; Lohkamp, B.; Scott, W. G.; Cowtan, K. Features and Development of Coot. *Acta Crystallogr. D Biol. Crystallogr.* **2010**, *66* (Pt 4), 486–501. https://doi.org/10.1107/S0907444910007493.

(9) Perrakis, A.; Sixma, T. K.; Wilson, K. S.; Lamzin, V. S. WARP: Improvement and Extension of Crystallographic Phases by Weighted Averaging of Multiple-Refined Dummy Atomic Models. *Acta Crystallogr. D Biol. Crystallogr.* **1997**, *53* (Pt 4), 448–455. https://doi.org/10.1107/S0907444997005696.

(10) Murshudov, G. N.; Vagin, A. A.; Dodson, E. J. Refinement of Macromolecular Structures by the Maximum-Likelihood Method. *Acta Crystallogr. D Biol. Crystallogr.* **1997**, *53* (Pt 3), 240–255. https://doi.org/10.1107/S0907444996012255.

(11) Tamura, K.; Stecher, G.; Kumar, S. MEGA11: Molecular Evolutionary Genetics Analysis Version 11. *Mol. Biol. Evol.* **2021**, *38* (7), 3022–3027. https://doi.org/10.1093/molbev/msab120.

(12) Robert, X.; Gouet, P. Deciphering Key Features in Protein Structures with the New ENDscript Server. *Nucleic Acids Res.* **2014**, *42* (W1), W320–W324. https://doi.org/10.1093/nar/gku316.

(13) Ashkenazy, H.; Abadi, S.; Martz, E.; Chay, O.; Mayrose, I.; Pupko, T.; Ben-Tal, N. ConSurf 2016: An Improved Methodology to Estimate and Visualize Evolutionary Conservation in Macromolecules. *Nucleic Acids Res.* **2016**, *44* (W1), W344–W350. https://doi.org/10.1093/nar/gkw408.

(14) Celniker, G.; Nimrod, G.; Ashkenazy, H.; Glaser, F.; Martz, E.; Mayrose, I.; Pupko, T.; Ben-Tal, N. ConSurf: Using Evolutionary Data to Raise Testable Hypotheses about Protein Function. *Isr. J. Chem.* **2013**, *53* (3–4), 199–206. https://doi.org/10.1002/ijch.201200096.

(15) Landau, M.; Mayrose, I.; Rosenberg, Y.; Glaser, F.; Martz, E.; Pupko, T.; Ben-Tal, N. ConSurf 2005: The Projection of Evolutionary Conservation Scores of Residues on Protein Structures. *Nucleic Acids Res.* **2005**, *33* (Web Server issue), W299–W302. https://doi.org/10.1093/nar/gki370.

(16) Glaser, F.; Pupko, T.; Paz, I.; Bell, R. E.; Bechor-Shental, D.; Martz, E.; Ben-Tal, N. ConSurf: Identification of Functional Regions in Proteins by Surface-Mapping of

Phylogenetic Information. *Bioinformatics* **2003**, *19* (1), 163–164. https://doi.org/10.1093/bioinformatics/19.1.163.
(17) Mordhorst, S.; Morinaka, B. I.; Vagstad, A. L.; Piel, J. Posttranslationally Acting Arginases Provide a Ribosomal Route to Non-Proteinogenic Ornithine Residues in Diverse Peptide Sequences. *Angew. Chem. Int. Ed.* **2020**, *59* (48), 21442–21447. https://doi.org/10.1002/anie.202008990.
(18) Ilies, M.; Di Costanzo, L.; Dowling, D. P.; Thorn, K. J.; Christianson, D. W. Binding of α,α-Disubstituted Amino Acids to Arginase Suggests New Avenues for Inhibitor Design. *J. Med. Chem.* **2011**, *54* (15), 5432–5443. https://doi.org/10.1021/jm200443b.
(19) *Origin(Pro), Version 2019 (9.60)*; OriginLab Corporation, Northampton, MA, USA.
(20) McGee, W. M.; McLuckey, S. A. The Ornithine Effect in Peptide Cation Dissociation. *J. Mass Spectrom.* **2013**, *48* (7), 856–861. https://doi.org/10.1002/jms.3233.
(21) Badmann, T.; Groll, M. Structures in Tetrahydrofolate Methylation in Desulfitobacterial Glycine Betaine Metabolism at Atomic Resolution. *ChemBioChem* **2020**, *21* (6), 776–779. https://doi.org/10.1002/cbic.201900515.
(22) Ticak, T.; Kountz, D. J.; Girosky, K. E.; Krzycki, J. A.; Ferguson, D. J. A Nonpyrrolysine Member of the Widely Distributed Trimethylamine Methyltransferase Family Is a Glycine Betaine Methyltransferase. *Proc. Natl. Acad. Sci. U. S. A.* **2014**, *111* (43), E4668–E4676. https://doi.org/10.1073/pnas.1409642111.
